# 5-Methylcytosine transferase NSUN2 drives NRF2-mediated ferroptosis resistance in non-small cell lung cancer

**DOI:** 10.1016/j.jbc.2024.106793

**Published:** 2024-02-24

**Authors:** Youming Chen, Zuli Jiang, Chenxing Zhang, Lindong Zhang, Huanxiang Chen, Nan Xiao, Lu Bai, Hongyang Liu, Junhu Wan

**Affiliations:** 1 Department of Clinical Laboratory, The First Affiliated Hospital of Zhengzhou University, Zhengzhou, Henan, China; 2 Department of Obstetrics and Gynecology, The Third Affiliated Hospital of Zhengzhou University, Zhengzhou, Henan, China; 3 School of Life Sciences, Zhengzhou University, Zhengzhou, Henan, China; 4 Department of General Surgery, Zhecheng People's Hospital, Shangqiu, Henan, China

**Keywords:** NSUN2, m5C modification, NRF2, ferroptosis, NSCLC

## Abstract

RNA 5-methylcytosine (m5C) is an abundant chemical modification in mammalian RNAs and plays crucial roles in regulating vital physiological and pathological processes, especially in cancer. However, the dysregulation of m5C and its underlying mechanisms in non-small cell lung cancer (NSCLC) remain unclear. Here we identified that NSUN2, a key RNA m5C methyltransferase, is highly expressed in NSCLC tumor tissue. We found elevated NSUN2 expression levels strongly correlate with tumor grade and size, predicting poor outcomes for NSCLC patients. Furthermore, RNA-seq and subsequent confirmation studies revealed the antioxidant-promoting transcription factor NRF2 is a target of NSUN2, and depleting NSUN2 decreases the expression of NRF2 and increases the sensitivity of NSCLC cells to ferroptosis activators both *in vitro* and *in vivo*. Intriguingly, the methylated-RIP-qPCR assay results indicated that NRF2 mRNA has a higher m5C level when NSUN2 is overexpressed in NSCLC cells but shows no significant changes in the NSUN2 methyltransferase-deficient group. Mechanistically, we confirmed that NSUN2 upregulates the expression of NRF2 by enhancing the stability of NRF2 mRNA through the m5C modification within its 5′UTR region recognized by the specific m5C reader protein YBX1, rather than influencing its translation. In subsequent rescue experiments, we show knocking down NRF2 diminished the proliferation, migration, and ferroptosis tolerance mediated by NSUN2 overexpression. In conclusion, our study unveils a novel regulatory mechanism in which NSUN2 sustains NRF2 expression through an m5C–YBX1–axis, suggesting that targeting NSUN2 and its regulated ferroptosis pathway might offer promising therapeutic strategies for NSCLC patients.

Lung cancer is the leading cause of cancer-related deaths worldwide (1, 2). The most prevalent histological type of lung cancer is non-small cell lung cancer (NSCLC), which constitutes about 80% of all lung cancer cases. NSCLC is recognized as a highly heterogeneous, aggressive, and relentlessly progressive disease with limited treatment options and poor survival rates (3, 4). Since most lung cancer symptoms manifest in advanced stages, significant efforts have been undertaken to enhance treatment efficacy. Over the past decade, a focus has been on developing molecular and histological techniques for cancer diagnosis and establishing targeted lung cancer therapies. However, traditional therapeutic approaches, such as chemotherapy, radiotherapy, targeted therapy, and immunotherapy, have not produced significant outcomes (5). Therefore, exploring the underlying molecular mechanisms of NSCLC progression and identifying novel treatment targets is crucial.

Posttranscriptional RNA modification occurs in all forms of RNA and is essential for every phase of its lifecycle. 5-methylcytosine (m5C) is a widely distributed posttranscriptional RNA modification primarily detected in tRNA and rRNA. It has also been demonstrated to exist in non-coding RNA and mRNA. NOP2/Sun RNA methyltransferase family member 2 (NSUN2) is the principal m5C methyltransferase responsible for the m5C modification of mammalian mRNA (6). A substantial body of evidence indicates that NSUN2 is highly expressed in multiple cancer types. The NSUN2-mediated RNA m5C modification contributes to the malignant phenotypes of tumors such as gastric cancer (7), cervical cancer (8), gallbladder carcinoma (9), esophageal squamous carcinoma (10). It is also associated with tumor proliferation, migration, and resistance to chemotherapy (11, 12). It is postulated that NSUN2-mediated m5C modification primarily impacts mRNA stability and translation efficiency, and the outcome of m5C modification might be closely tied to the methylation site or the specific modified mRNA. For instance, m5C modification by NSUN2 enhances HDGF1 mRNA stability in bladder cancer cells (13). The m5C modification of the 3′ UTR region of NSUN2-mediated cyclin-dependent kinase 1 mRNA boosts its translation by increasing the assembly of ribosomes on cyclin-dependent kinase 1 mRNA (14, 15). NSUN2-mediated m5C modification of the QSOX1 mRNA CDS also elevates its translation efficiency (12). However, the exact role of NSUN2 in NSCLC progression remains unreported, and the underlying molecular mechanisms are yet to be understood.

Ferroptosis, as a novel therapeutic approach for anticancer treatment, including therapy for NSCLC, is garnering significant attention (16, 17). Ferroptosis is governed by the balance between intracellular oxidation and antioxidation systems (18) and mainly comprises three elements: iron overload, lipid metabolism, and the glutathione peroxidase 4 (GSH/GPX4) pathway (19). Excessive free iron (Fe2+) instigates the generation of reactive oxygen species (ROS) through the Fenton reaction, leading to cell death (20). FTH1, the heavy chain subunit of ferritin, catalyzes the oxidation of Fe2+ to Fe3+ for storage, subsequently reducing the concentration of free iron within cells (21). The selenium-containing enzyme GPX4 is acknowledged as the central antioxidant in ferroptosis (22). Interestingly, nuclear factor erythroid 2-related factor 2 (NRF2) has been shown to translocate to the nucleus and enhance the transcription of GPX4, FTH1, and other antioxidants, resulting in the inhibition of ferroptosis under oxidative stress conditions (23). A constantly activated NRF2 mutant has been established to be responsible for resistance to chemotherapeutic drugs and radiotherapy (24, 25, 26). Additionally, elevated NRF2 expression levels in NSCLC correlate with unfavorable outcomes (27). Targeting NRF2 to enhance chemotherapeutic efficacy has proven effective in cell cultures and NSCLC xenograft mouse models (28). However, there are limited reports and studies concerning ferroptosis-based therapy resistance in NSCLC involving NSUN2.

Our previous study reported that NSUN2 is highly expressed and predicts a worse overall survival rate for NSCLC patients. We identified NRF2 as the target of NSUN2, and knocking down NRF2 reversed the ferroptosis alleviation and malignant phenotype mediated by NSUN2. NSUN2-mediated m5C modification of the NRF2 mRNA 5′UTR region enhances NRF2 mRNA stability in an m5C-YBX1–dependent manner. Thus, our work highlighted the significant role of the NSUN2–NRF2–YBX1 axis in regulating lung cancer cell ferroptosis and suggested its potential as a therapeutic target for NSCLC.

## Results

### NSUN2 is elevated in NSCLC tissues and cells

**Figure 1:**
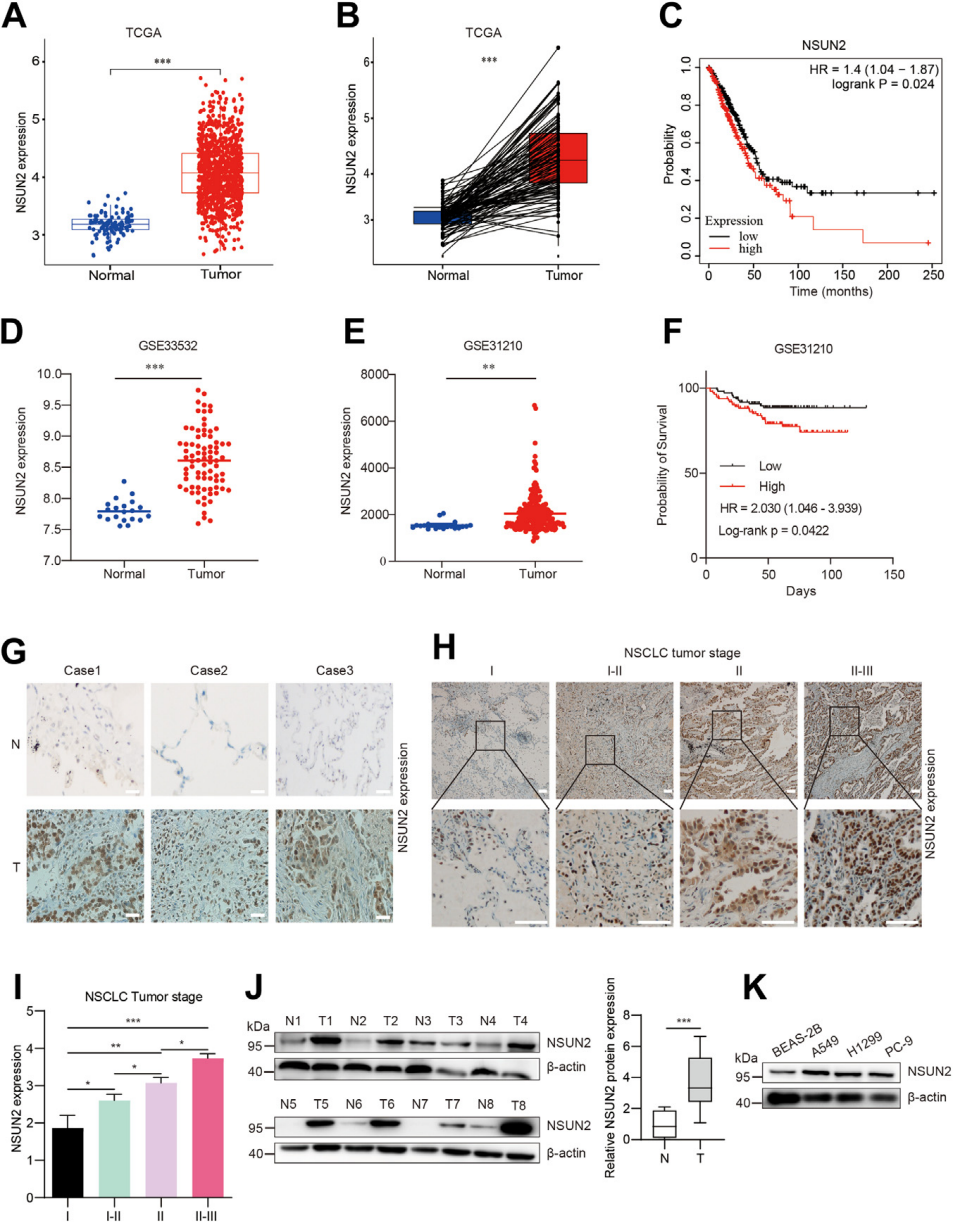
**NSUN2 is highly expressed in non-small cell lung carcinoma cells and tissues.***A*, the NSUN2 expression level between LUAD (Lung Adenocarcinoma) and normal tissues was analyzed using the TCGA database. *B*, the NSUN2 expression in the paired LUAD tumors was analyzed by the TCGA database. *C*, Kaplan-Meier analysis of the TCGA-LUAD dataset showing high NSUN2 expression predicted worse overall survival. *p* value (*p* = 0.024) was determined by the log-rank test. *D* and *E*, NSUN2 expression differences between LUAD and normal tissues were analyzed using the GEO database GSE33532 and GSE31210. *F*, Kaplan-Meier analysis of GEO database GSE31210 showing high NSUN2 expression predicted worse overall survival. *p* value (*p* = 0.0422) was determined by the log-rank test. *G*, IHC analysis of NSUN2 expression in the paired normal and LUAD tissues. Scale bar represents 100 μm. *H*, IHC analysis of NSUN2 expression in LUAD at I, I-II, II, and II-III stages; the IHC score was quantitated in (*I*). The data are expressed as mean ± SD. Scale bar represents 100 μm. *J*, protein levels of NSUN2 in 8-paired lung adenocarcinoma and adjacent tissues were analyzed by Western blot. The *gray* value of Western blot was analyzed by ImageJ software, and the statistical analysis was performed (*right*). *K*, Western blot of NSUN2 expression in NSCLC cell lines. ∗*p* < 0.05; ∗∗*p* < 0.01; ∗∗∗*p* < 0.001. IHC, immunohistochemistry; NSCLC, non-small cell lung cancer; NSUN2, NOP2/Sun RNA methyltransferase family member 2; TCGA, The Cancer Genome Atlas.

**Supporting Figure S1:**
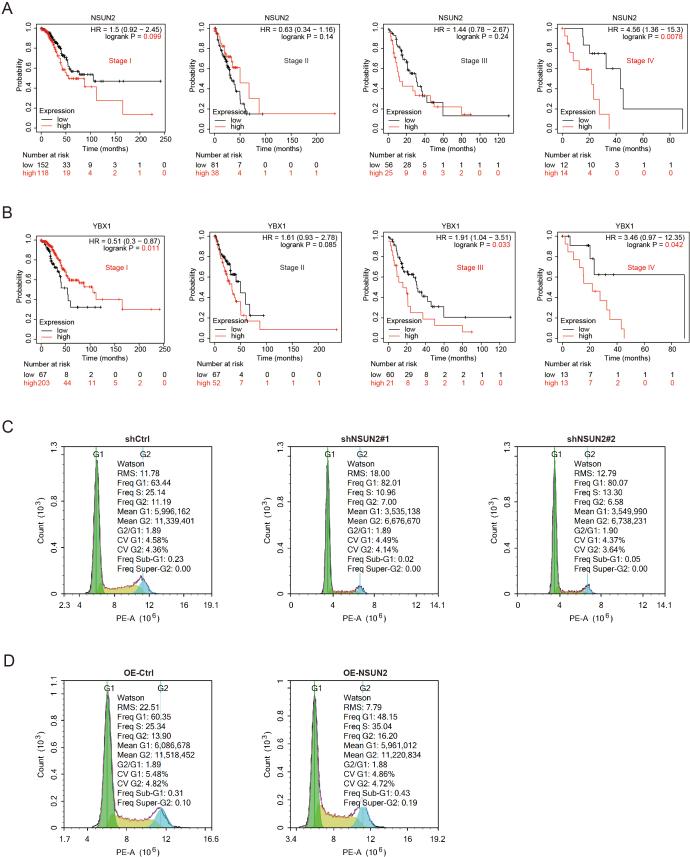
*A*, the Kaplan-Meier analysis of NSUN2 in tumor stages of LUAD patients. *B*, the Kaplan-Meier analysis of YBX1 in tumor stages of LUAD patients. *C*, the cell cycle of NSUN2 knocking down or overexpressing A549 cells (*D*) was detected by flow cytometry. ∗*p* < 0.05; ∗∗*p* < 0.01; ∗∗∗*p* < 0.001.

**Supporting Figure S2:**
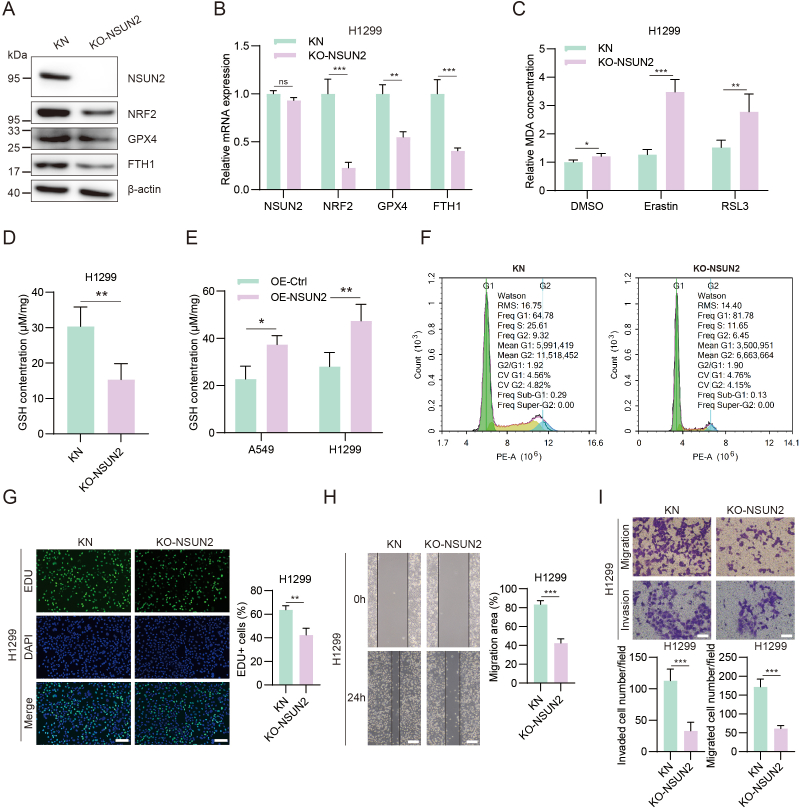
*A*, the western blot analysis of NSUN2 knockout efficiency performed by CRISPR/Cas9 technology and protein expression of NRF2, GPX4, and FTH1 in NSUN2 KO H1299 cells. *B*, cell viability and MDA levels (*C*) of NSUN2 knockout H1299 cells. *D*, the GSH concentration in NSUN2 knockout and overexpression of H1299 cells (*E*). *F*, cell cycle of NSUN2 knockout H1299 cells was determined by flow cytometry. *G*, the proliferation ability of NSUN2 knockout H1299 cell was detected by EdU incorporation assay. Scale bar represents 100 μm. *H*, the migration and invasion abilities of NSUN2 knockout H1299 cells were measured by wound healing assay and transwell assays (*I*). Data are mean ± SD of three independent experiments. Scale bar represents 100 μm ns, not significant; ∗*p* < 0.05; ∗∗*p* < 0.01; ∗∗∗*p* < 0.001.

To evaluate the expression profile of NSUN2 in human NSCLC tumors and normal tissue, we first analyzed RNA-seq data from the TCGA and GEO databases (GSE33532, GSE31210). We found that NSUN2 was highly expressed in NSCLC tissues (Fig. 1, *A*, *D* and *E*) and in paired tumors (Fig. 1*B*), compared to normal tissues. Next, we examined the transcriptome data and the corresponding survival information from these databases. We determined that a higher expression of NSUN2 predicted a worse prognosis for patients with NSCLC (Fig. 1, *C* and *F*), especially in the stage I and IV (Fig. S1*A*). Additionally, we used immunohistochemistry (IHC) and Western blot assays to detect NSUN2 expression in normal and NSCLC tumor tissues collected from The First Affiliated Hospital of Zhengzhou University. Before the IHC staining, we have been performed the Western blot analysis in NSUN2 KO cell line as shown in Fig. S2*A*. In line with the results from the TCGA and GEO cohorts, high NSUN2 protein expression was more frequently observed in NSCLC tumor tissues than in normal tissues (Fig. 1*G*). NSUN2 protein expression also showed a positive correlation with the NSCLC tumor stage (Fig. 1, *H* and *I*). We analyzed the correlation between NSUN2 and clinicopathological parameters based on NSUN2 expression and clinical data (Table 1). The results indicated that high levels of NSUN2 expression were positively correlated with NSCLC tumor grade and size. However, there was no discernible correlation between NSUN2 expression and gender, age, or tumor-distant metastasis. Our Western blot analysis further confirmed the higher NSUN2 expression in tumors than in normal tissues (Fig. 1*J*). Moreover, as illustrated in Figure 1*K*, NSUN2 was more highly expressed in several NSCLC cell lines, such as H1299, A549, and PC-9 cells, than in the normal human lung epithelial cell line BEAS-2B. And we found that NSUN2 has a relative middle expression in A549 and H1299 cell lines among all the NSCLC cell lines according to Cancer Cell Line Encyclopedia (CCLE) database recorded (Table S1). These findings suggest that NSUN2 is upregulated in NSCLC tumors, and a higher NSUN2 expression correlates with a worse prognosis.

**Table 1:** Correlation of NSUN2 expression and clinical features in patients with LUAD

Variables	NSUN2 expression		*p* Value
	Low (n = 45)	High (n = 75)	
Gender			0.161
Male	14	33	
Female	31	42	
Age (years)			0.767
<60	15	27	
≥60	30	48	
Tumor size (cm)			**0.018∗**
<3	40	58	
≥3	5	17	
Tumor number			0.449
Signal	41	71	
Multiple	4	4	
TNM stage			**0.0008∗∗∗**
I	10	5	
I–II	17	18	
II	18	37	
II-III	0	15	
Vascular invasion			0.88
No	44	73	
Yes	1	2	

The median expression level of NSUN2 was used as the cutoff value. ∗*p* < 0.05, ∗∗*p* < 0.01, ∗∗∗*p* < 0.001.

### NSUN2 deficiency significantly represses NSCLC progression *in vitro*

**Figure 2:**
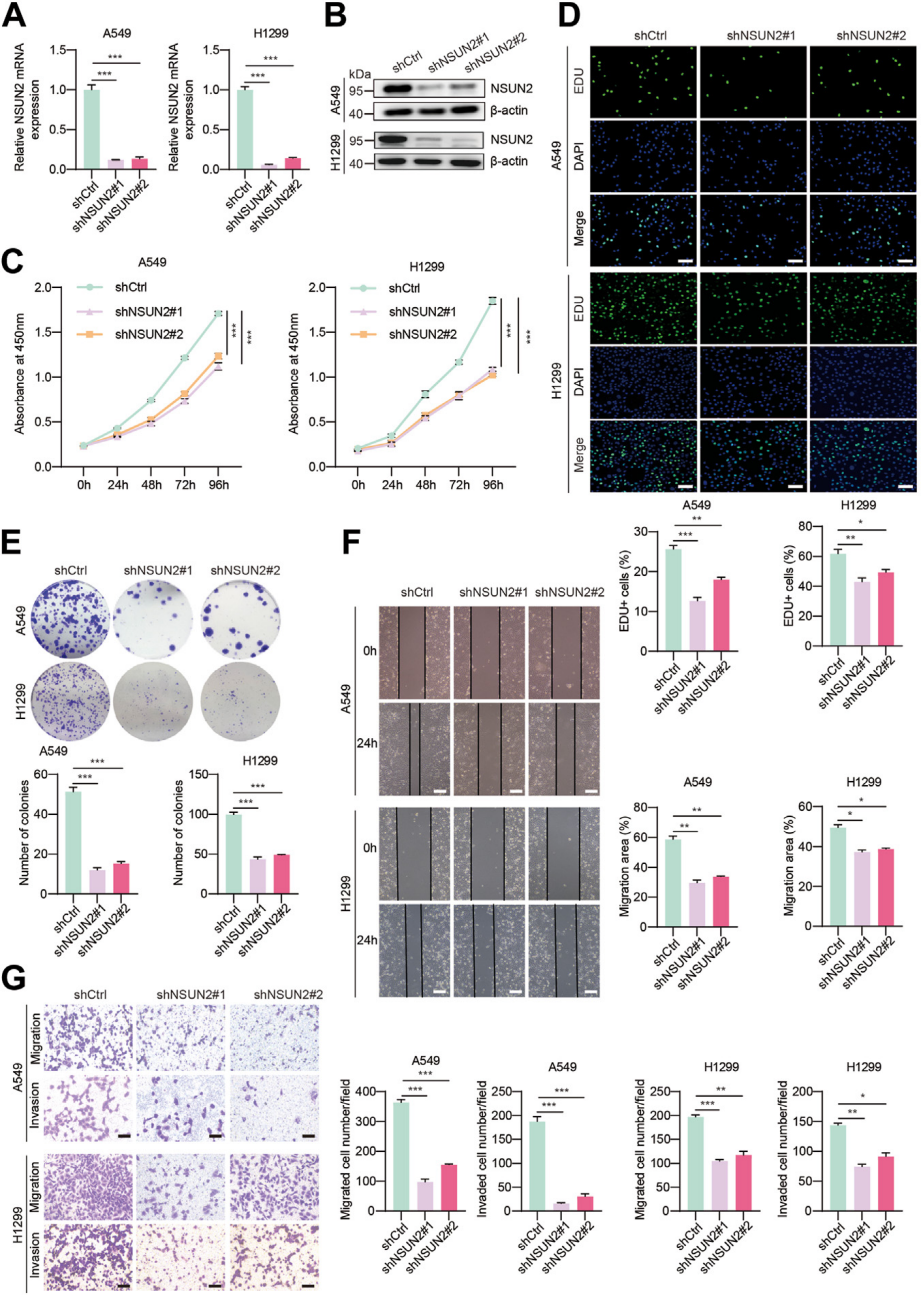
**NSUN2 deficiency significantly represses NSCLC progression *in vitro*.***A*, NSUN2 mRNA expression was analyzed by RT-qPCR after knockdown of NSUN2 with shRNA. *B*, relative protein levels of NSUN2 after inhibition were determined by Western blotting. *C*, cell viability of A549 and H1299 cells was assessed by CCK-8 assay. *D*, cell proliferation after the knockdown of NSUN2 was detected by the EDU assay (Magnification 100×. Scale bar represents 100 μm). The *top* are representative images and at the *bottom* are the statistical analysis graphs. *E*, colony formation assays were performed in the A549 and H1299 cell lines in which NSUN2 was knocked down. The *top* are representative images and at the bottom are the statistical analysis graphs. *F*, migration abilities of A549 and H1299 cells after depletion of NSUN2 were evaluated by wound-healing assay. The *right* is the representative images and the *bottom-right* is the statistical analysis. *G*, migration and invasion abilities of A549 and H1299 cells after NSUN2 inhibition were evaluated by transwell assays. The *left* is representative images and the *right* is the statistical analysis graphs. Scale bar represents 100 μm. ∗*p* < 0.05; ∗∗*p* < 0.01; ∗∗∗*p* < 0.001. NSCLC, non-small cell lung cancer; NSUN2, NOP2/Sun RNA methyltransferase family member 2; RT-qPCR, real time quantitative PCR.

To evaluate the biological role of NSUN2 in NSCLC, we initially selected the human NSCLC cell lines A549 and H1299 for further functional investigation. Firstly, we conducted *in vitro* functional assays to explore the role of *NSUN2* in NSCLC progression. We silenced *NSUN2* in A549 and H1299 cells using lentiviral transfection. The knockdown efficiency was confirmed by real time quantitative PCR (RT-qPCR) (Fig. 2*A*) and Western blot (Fig. 2*B*). As demonstrated in the CCK8 assays, suppressing *NSUN2* reduced the viability of lung cancer cells compared to their respective controls over time (Fig. 2*C*). We then conducted EdU and colony formation assays to further examine the functional role of *NSUN2* in NSCLC cell proliferation. Interestingly, under *NSUN2* inhibition, we observed fewer proliferative cells double-labeled with EdU (Fig. 2*D*) and decreased number of colonies (Fig. 2*E*), suggesting that *NSUN2* deficiency markedly attenuates the proliferation capability of NSCLC cells. In mechanism, we found that the cell cycle was greatly arrested after NSUN2 deficiency when the cell was measured by flow cytometry (Fig. S1*C*). Since the primary cause of death in cancer is often metastasis and deregulation of cell migration during cancer progression can determine the potential of cancer cells to invade adjacent tissues and form metastases, we assessed the effect of NSUN2 on NSCLC cell migration and invasion. The wound-healing assay results indicated that *NSUN2* inhibition significantly reduced the mobility of A549 and H1299 cells in comparison to the control group (Fig. 2*F*). Consistently, with *NSUN2* knocked down, the migration ability of H1299 and A549 cells also diminished, as observed in the transwell assays (Fig. 2*G*). Recognizing that invasive migration is crucial for processes such as angiogenesis, embryonic development, immune responses, metastasis, and cancer invasion, we performed transwell assays with Matrigel to assess cellular invasion capability. The results showed that silencing *NSUN2* reduced the number of invasive cells (Fig. 2*G*). Overall, the findings indicate that knocking down *NSUN2* adversely impacts the progression of NSCLC *in vitro*, suggesting that NSUN2 may play an oncogenic role.

What’s more, to further confirmed the oncogenic role of NSUN2 in NSCLC, we established *NSUN2*-KO H1299 cell line clone by using CRISPR/Cas9 technology. After the testification of knockout efficiency (Fig. S2*A*), we also conducted several cell phenotype experiments and found that the cell cycle of H1299 was greatly arrested (Fig. S2*F*), the proliferation ability was inhibited (Fig. S2*G*), the migration and invasion abilities were blocked significantly (Fig. S2, *H* and *I*) after *NSUN2* knockout, which was further provided the evidence for NSUN2 tumor-promoting role in NSCLC.

### NSUN2 overexpression promotes NSCLC progression *in vitro*

**Figure 3:**
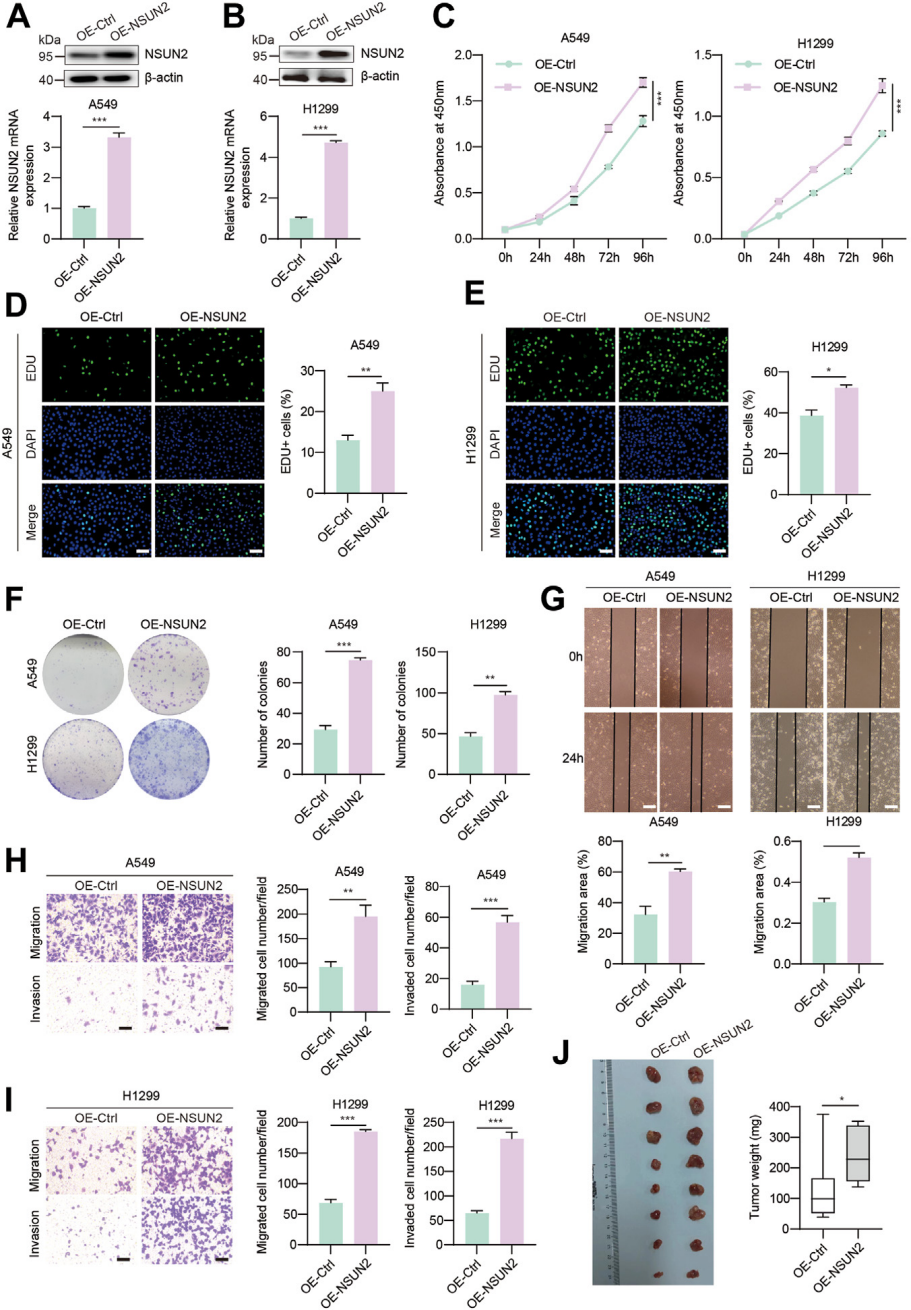
**NSUN2 enhances NSCLC cell proliferation *in vitro* and *in vivo*.***A* and *B*, the relative protein and mRNA levels of NSUN2 overexpression in A549 and H1299 cells were determined by Western blotting and RT-qPCR, respectively. *C*, CCK8 assay and (*D* and *E*) EdU assay were conducted to determine the cell proliferation ability *in vitro*. *F*, colony formation assays were performed in A549 and H1299 cell lines overexpressing NSUN2. The *right* is the representative image and the *left* is the statistical analysis. *G*, the results of the wound-healing assay confirmed the cell migration ability after NSUN2 overexpression. The *top* is the representative image and the *bottom* is the statistical analysis. *H* and *I*, transwell assay indicated the migration and invasive ability of A549 and H1299 cells when overexpressing NSUN2. The *right* is the representative image and the *left* is the statistical analysis. *J*, effects of overexpressing NSUN2 on tumor weight and volume in the subcutaneous xenograft nude mouse model. Data are presented as means ± SD. Scale bar represents 100 μm. ∗*p* < 0.05; ∗∗*p* < 0.01; ∗∗∗*p* < 0.001. NSCLC, non-small cell lung cancer; NSUN2, NOP2/Sun RNA methyltransferase family member 2; RT-qPCR, real time quantitative PCR.

To further confirm the regulatory function of NSUN2 in NSCLC progression, we stably constructed NSCLC cell lines overexpressing NSUN2 using lentiviral infection. The upregulation of *NSUN2* expression was verified by RT-qPCR and Western blotting (Fig. 3, *A* and *B*). Cell counting kit (CCK)-8 assays were then conducted to determine the role of NSUN2 in the growth of lung cancer cells. Consistent with the shRNA-mediated knockdown results, *NSUN2* overexpression significantly enhanced the viability of NSCLC cell lines (A549 and H1299) (Fig. 3*C*). Similarly, EdU proliferation assays revealed that *NSUN2* overexpression increased the proportion of cells incorporating EdU (Fig. 3, *D* and *E*). Additionally, the number of cell colonies was notably elevated in the *NSUN2* overexpression groups compared to the control (Fig. 3*F*). Interestingly, we found that the cell cycle times of *NSUN2* overexpression cell was shortened compared to the control (Fig. S1*D*), which indicates that NSUN2 promotes NSCLC cell proliferation by modulating cell cycle. Wound healing and transwell assays were subsequently performed to gauge the effects of *NSUN2* overexpression on cell migration and invasion. As shown in Figure 3*G*, NSUN2 significantly accelerated wound closure compared to control cells. Furthermore, transwell assays indicated that the number of lung cancer cells that migrated through the membrane and their invasive capacity was substantially augmented in the *NSUN2* overexpressed groups (Fig. 3, *H* and *I*). To further examine the effects of NSUN2 on NSCLC *in vivo*, we established a mouse NSCLC xenograft model. As depicted in Figure 3*J*, the average tumor volume of the harvested tumors from the *NSUN2* overexpressed group was larger than that from the NC group. The average tumor weights for the NC and *NSUN2* overexpression groups were 0.239 g and 0.050 g, respectively. These results suggest that ectopic expression of *NSUN2* promotes NSCLC tumorigenesis by amplifying cell proliferation, migration, and invasion capabilities.

### NSUN2 deficiency sensitizes NSCLC cells to ferroptosis

**Figure 4:**
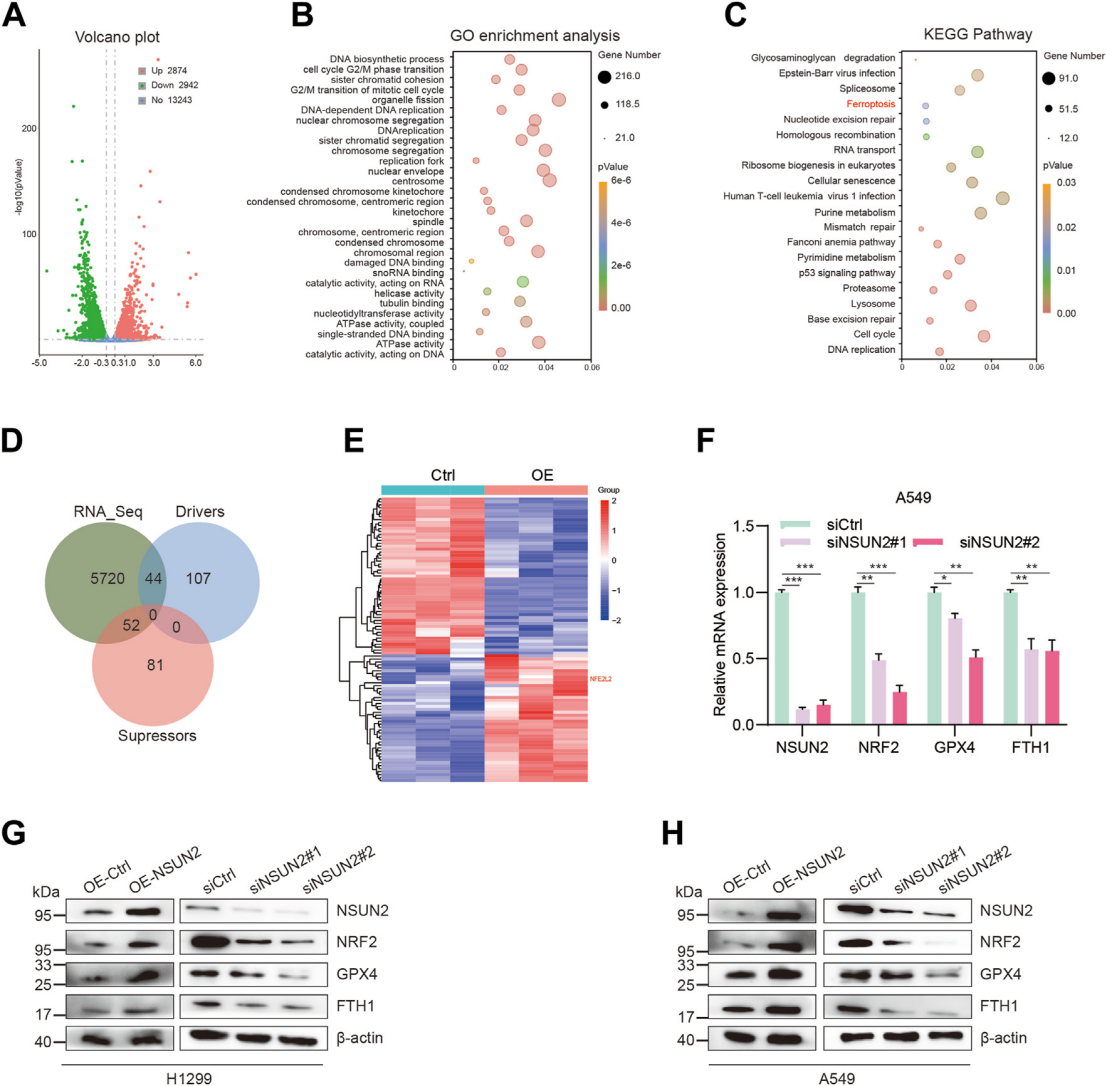
**RNA-seq and bioinformatic analysis in NSUN2 overexpression of A549 cell.***A*, volcanic plot of different mRNAs in A549 cells overexpressing NSUN2. *B*, gene ontology and KEGG (Kyoto Encyclopedia of Genes and Genomes) pathways analysis (*C*) of the differentially expressed mRNAs described in (*A*). *D*, venn diagram showing the potential targets of NSUN2 in ferroptosis *via* the combination with ferroptosis database FerrDb. *E*, the heatmap displayed the expression ratios of different expressed ferroptosis-related genes from RNA-seq analysis. *F*, the mRNA levels of NRF2, GPX4, and FTH1 RT-qPCR, and (*G* and *H*) protein expression levels by Western blot. Data are mean ± SD. ∗*p* < 0.05, ∗∗*p* < 0.01, ∗∗∗*p* < 0.001. NSUN2, NOP2/Sun RNA methyltransferase family member 2; NRF2, nuclear factor erythroid 2-related factor 2; RT-qPCR, real time quantitative PCR.

To identify the target gene associated with NSUN2-regulated cancer progression, we conducted RNA-seq on *NSUN2*-overexpressing A549 cells. In total, 5816 differentially expressed genes (DEGs) were identified through volcano plot analysis (Fig. 4*A*). Gene ontology and Kyoto Encyclopedia of Genes and Genomes analyses revealed that these DEGs were linked to several tumor-related pathways, such as cell cycle, DNA replication, DNA damage repair, cellular senescence, p53 signaling pathway, and ferroptosis, among others (Fig. 4, *B* and *C*). It is now understood that the inhibition of ferroptosis is essential for sustaining tumor proliferation (29). Ferroptosis has been explored as an alternative strategy to target tumor cells resistant to drugs, potentially sparing normal cells (30). To delineate the relationship between NSUN2 and ferroptosis, we screened the ferroptosis-related genes using the FerrDb database, displaying the overlap in a Venn diagram (Fig. 4*D*) and presenting the genes in a heatmap (Fig. 4*E*). In our RNA-seq results, we observed that encodes the protein NRF2 (*NFE2L2*) was one of the most significantly upregulated genes. Additionally, we noted the upregulation of its targets, *GPX4* and *FTH1* mRNA, following *NSUN2* overexpression. And we observed that, the expression of *NRF2*, *GPX4*, and *FTH1* were also attenuated after *NSUN2* knocked down, as confirmed by RT-qPCR (Fig. 4*F*) and Western blot analysis (Fig. 4, *G* and *H*).

**Figure 5:**
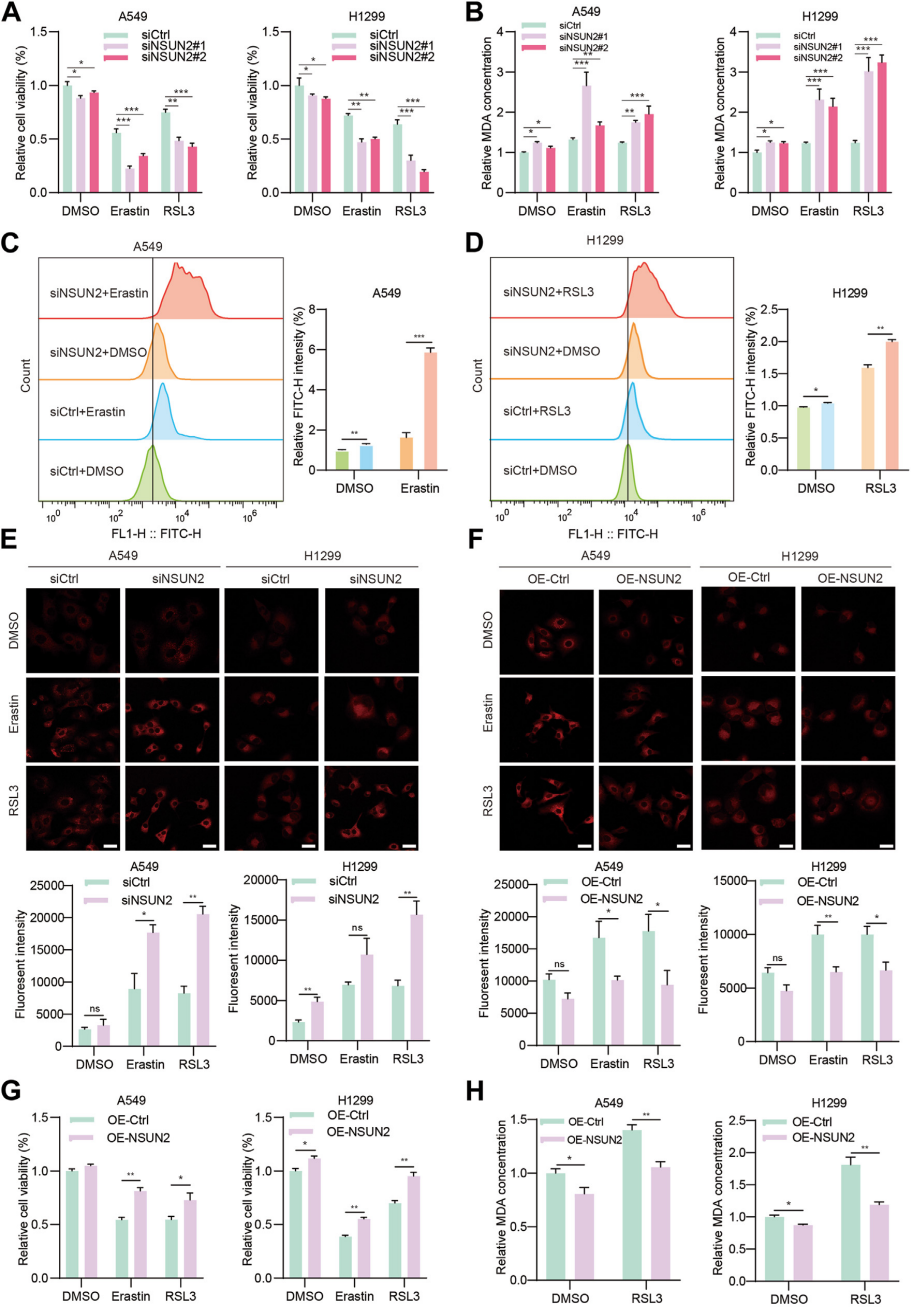
**NSUN2 deficiency sensitizes NSCLC cells to ferroptosis.***A*, cell viability was assessed following treatment with erastin and RSL3 in NSUN2 knockdown NSCLC cells. *B*, lipid peroxidation levels in NSUN2 knocking down A549 and H1299 cells were determined by MDA assay. *C* and *D*, lipid ROS accumulation was analyzed by flow cytometry with C11-BODIPY staining. The *left* is the representative image and the *right* is the statistical analysis. *E* and *F*, the intracellular Fe2+ was measured by iron detection assay. The *upper* are the representative images and the under are the statistical analysis. Scale bar represents 20 μm. *G*, the cell viability and MDA levels (*H*) were assessed following NSUN2 overexpression in A549 and H1299 cells. Data are mean ± SD. ns, not significant; ∗*p* < 0.05; ∗∗*p* < 0.01; ∗∗∗*p* < 0.001. NSCLC, non-small cell lung cancer; NSUN2, NOP2/Sun RNA methyltransferase family member 2.

Ferroptosis-based cancer treatment is emerging as a novel therapeutic approach in NSCLC. Thus, to investigate the functional role of NSUN2 in the ferroptosis of NSCLC cells, we incubated NSCLC cells with overexpressed or knocked down *NSUN2* with erastin or RSL3 for 12 h. Subsequently, we examined ferroptosis-specific markers, including cell viability, malondialdehyde (MDA) level, lipid ROS level, intracellular free Fe2+ level, and glutathione (GSH) concentration. Interestingly, upon knocking down *NSUN2*, both A549 and H1299 cells exhibited reduced cell viability (Fig. 5*A*), increased lipid-peroxidation by-product MDA (Figs. 5*B* and S2*C*) levels, and elevated intracellular lipid ROS levels (Fig. 5, *C* and *D*) after erastin and RSL3 treatment. Additionally, the levels of intracellular free Fe2+ showed a modest rise post-*NSUN2* knockdown (Fig. 5*E*), and the levels of GSH concentration showed a significant decrease after *NSUN2* knockout (Fig. S2*D*), suggesting a general loss in the cell's anti-ferroptosis capacity. To further corroborate our findings, we examined these ferroptosis markers in NSCLC cells with stable *NSUN2* overexpression. Predictably, heightened *NSUN2* expression could partially mitigate the ferroptosis induced by erastin and RSL3. Cells in the *NSUN2* overexpression group exhibited a reduced intracellular Fe2+ concentration (Fig. 5*F*), increased cell viability (Fig. 5*G*), diminished MDA levels (Fig. 5*H*), and enhanced GSH concentration (Fig. S2*E*) compared to the control. This implies that NSUN2 might exert an anti-ferroptosis effect. In conclusion, these findings demonstrate that NSCLC cells are susceptible to erastin and RSL3 and that the deficiency of *NSUN2* can significantly amplify this effect.

### NSUN2 increases NSCLC ferroptosis-resistance capacity by inducing NRF2 activation

**Figure 6:**
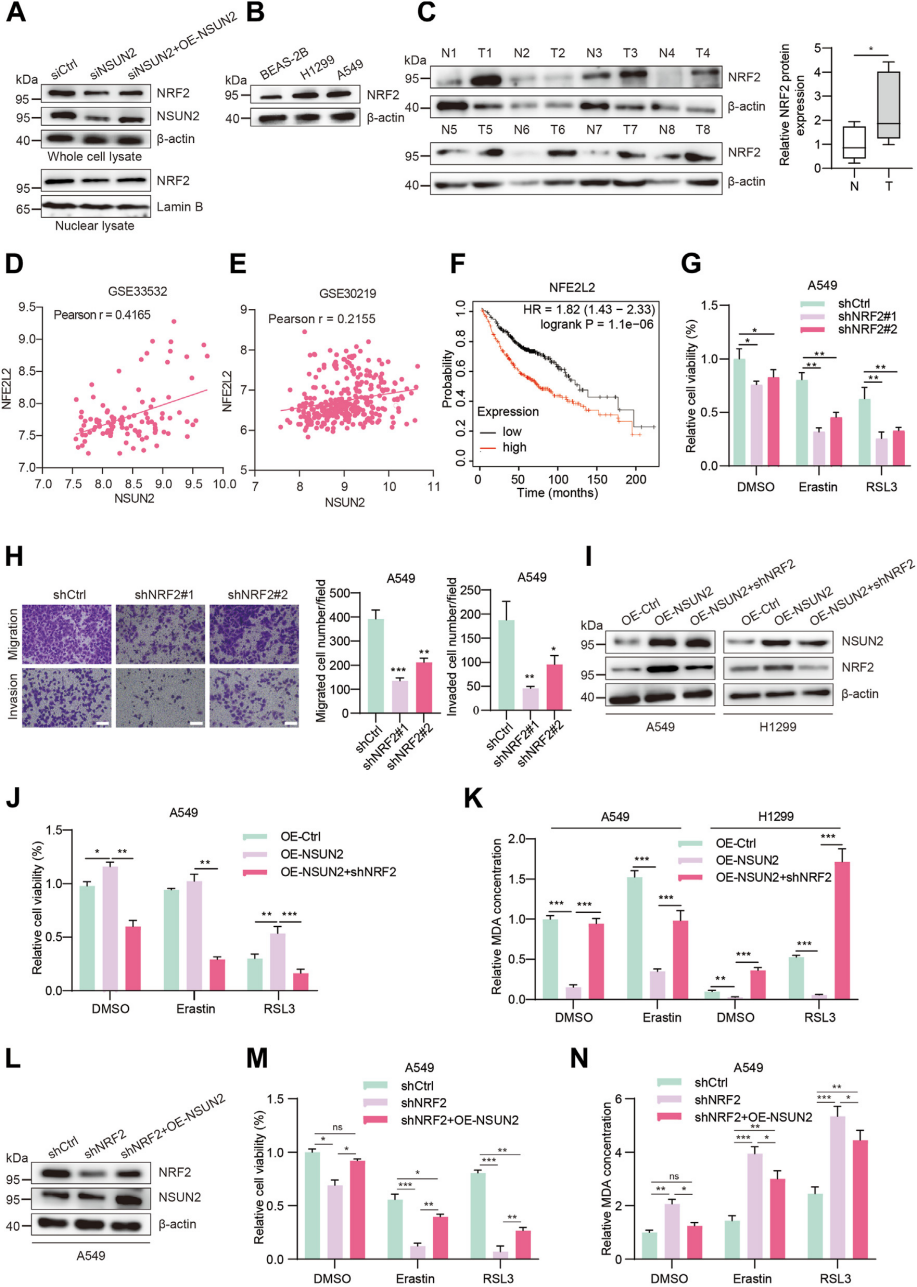
**NSUN2 increases NSCLC ferroptosis-resistance capacity by inducing NRF2 activation.***A*, Western blot of NRF2 protein abundance in the whole cell and nucleus of NSUN2 knockdown A549 cells self-rescued by overexpressing NSUN2. *B*, Western blot of NRF2 protein expression in NSCLC cell lines and paired clinical samples (*C*); the *gray* value of Western blot is quantified by ImageJ software and statistical analysis was performed (*right*). Data are shown as mean ± SD. *D*, the correlation of NRF2 mRNA and NSUN2 in RNA-seq analysis from GEO database GSE33532 (*D*) and GSE30219 (*E*). *F*, Kaplan-Meier analysis of NSCLC patients based on NRF2 expression in the TCGA database. *p* value (*p* = 1.1e-06) was analyzed by log-rank test. *G*, the relative cell viability of A549 cells after NRF2 inhibition. *H*, the migration and invasion capabilities of A549 cells after NRF2 inhibition were detected by transwell assays. The *left* are representative images and the *right* are the statistical analysis graphs. Scale bar represents 100 μm. *I*, Western blot of NRF2 expression in NSUN2-overexpressing A549 and H1299 cells followed with NRF2 knockdown by shNRF2, the relative cell viability (*J*), and MDA levels were determined to evaluate the progression of ferroptosis (*K*). *L*, Western blot of NRF2 expression in NRF2-knockdown A549 cells followed with NSUN2 overexpression; the relative cell viability (*M*) and MDA levels (*N*) were determined to evaluate the ferroptosis. Data are shown as mean ± SD. shNRF2 means the shNRF2#1. ns, not significant; ∗*p* < 0.05; ∗∗*p* < 0.01; ∗∗∗*p* < 0.001. NRF2, nuclear factor erythroid 2-related factor 2; NSCLC, non-small cell lung cancer; NSUN2, NOP2/Sun RNA methyltransferase family member 2; TCGA, The Cancer Genome Atlas.

*NFE2L2* was among the most notably upregulated genes in our RNA-seq results, drawing our attention. Constitutive activation of NRF2 provides cells with a malignant phenotype, and efforts to develop anti-tumor drugs targeting NRF2 activation have been ongoing in recent years (31). As observed in our rescue experiment, NRF2 activation was ablated when we knocked down *NSUN2* in the overexpression-stable cells. The nuclear abundance of the NRF2 protein decreased upon *NSUN2* knockdown, but this reduction could be reversed by *NSUN2* overexpression (Fig. 6*A*). And we also observed the expression of *NFE2L2* was inhibited both in protein and mRNA levels after *NSUN2* was knocked out in H1299 cells, as well as the expression of *GPX4* and *FTH1* (Fig. S2, *A* and *B*). Given NRF2's crucial role in driving NSCLC tumorigenesis, metastasis, and drug resistance, our results also confirmed that NRF2 maintains a higher expression level in NSCLC cell lines than in normal lung epithelial cells (Fig. 6*B*). Additionally, NRF2 protein expression is more pronounced in NSCLC tumors than in their matched normal tissues (Fig. 6*C*). It positively correlates with NSUN2 protein expression (Fig. 6, *D* and *E*) and predicts a poorer overall survival rate (Fig. 6*F*). After silencing *NFE2L2*, cells exhibit increased sensitivity to ferroptosis inducers (Fig. 6*G*), as well as impaired migration and invasion abilities *in vitro* (Fig. 6*H*).

**Supporting Figure S3:**
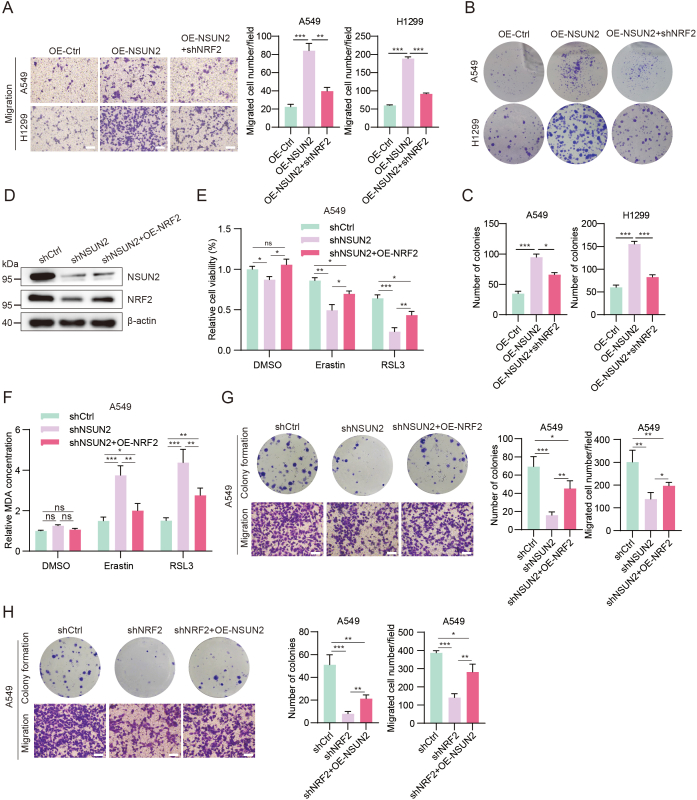
*A*, the migration ability of NSUN2 overexpression with or without NRF2 knockdown A549 and H1299 cells was measured by transwell assay. *B* and *C*, the proliferation ability of NSUN2 overexpression with or without NRF2 knockdown A549 and H1299 cells was measured by clonal formation assay. *D*, the western blot analysis of NSUN2 and NRF2 expression in NSUN2 knockdown with or without NRF2 overexpression A549 cells, and the cell viability (*E*) and MDA levels (*F*) were determined to evaluate the ferroptosis. *G*, the proliferation and migration abilities of NSUN2 knockdown with or without NRF2 overexpression A549 cells were confirmed by clonal formation and transwell assays, respectively. *H*, the proliferation and migration abilities of NRF2 deficiency or rescued by NSUN2 overexpression A549 cell were confirmed by clonal formation and transwell assays, respectively. Data are mean ± SD of three independent experiments. Scale bar represents 100 μm. ns, not significant; ∗*p* < 0.05; ∗∗*p* < 0.01; ∗∗∗*p* < 0.001.

To further determine whether NSUN2 promotes NSCLC tumorigenesis and ferroptosis tolerance through NRF2 regulation, we undertook a functional recovery experiment. Upon overexpressing NSUN2, we suppressed *NFE2L2* expression using shRNA, and the knockdown efficiency was confirmed by Western blot (Fig. 6*I*). Notably, reducing NRF2 expression decreased the viability of NSCLC cells and substantially raised the levels of MDA, a byproduct of lipid peroxidation (Fig. 6, *J* and *K*), which indicts the ferroptosis tolerance capability decrease. In addition, the proliferation and migration abilities of *NSUN2* overexpressing cells also significantly diminished after NRF2 knocking down (Fig. S3, *A*–*C*). Similarly, we upregulated the expression of NRF2 in *NSUN2* knocking down A549 cells and confirmed the efficiency by Western blot (Fig. S3*D*), and we found that NRF2 upregulation increased the cell viability (Fig. S3*E*) and MDA levels (Fig. S3*F*) after the administration of ferroptosis inducers. It was no doubt that NRF2 upregulation also rescued the *NSUN2* deficiency–mediated cell proliferation and migration inhibition (Fig. S3*G*). In turn, we also observed that overexpressing NSUN2 could partly reverse the blockaded NRF2 expression in NRF2-knockdown A549 cells (Fig. 6*L*), as well as the repressed cell viability (Fig. 6*M*) and upregulated MDA levels (Fig. 6*N*), which results suggest that NRF2 is a potential target of NSUN2, and NSUN2 may bolster NSCLC ferroptosis tolerance by enhancing NRF2 protein expression *in vitro*.

### NRF2 activation depends on NSUN2-medicated RNA m5C modification

**Figure 7:**
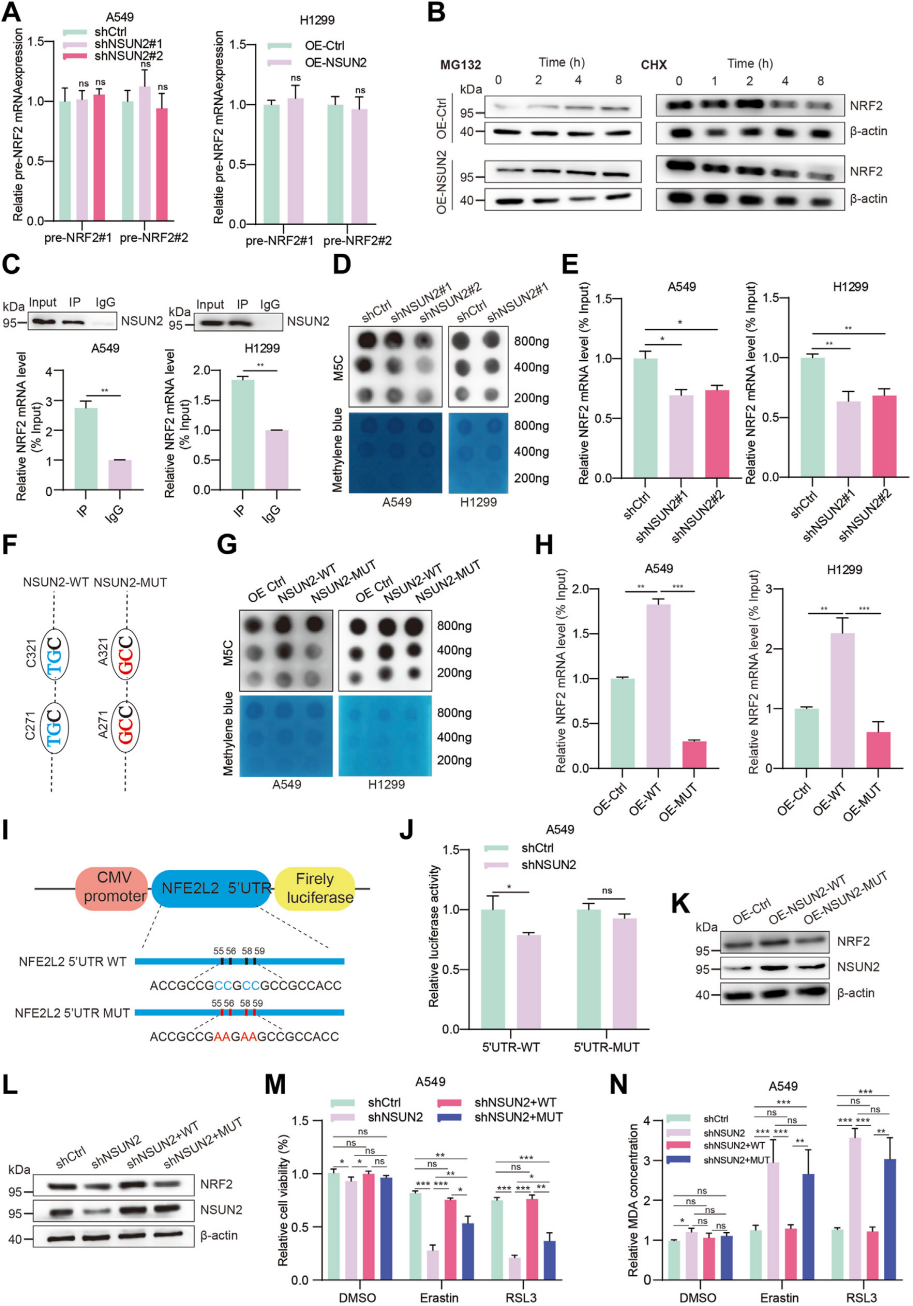
**NSUN2 promotes NRF2 mRNA m5C modification formation.***A*, pre-NRF2 mRNA levels in NSUN2 knockdown (*left* graph) and overexpression (*right* graph) NSCLC cells. *B*, Western blot of NRF2 protein expression after MG132 (*left*) and CHX (*right*) treatment for the indicated time. *C*, A549 H1299 RNA immunoprecipitation of NSUN2 and NRF2 mRNA was performed in A549 and H1299 cells, with IgG as the control. *D*, The m5C dot blot assay was used to detect the levels of m5C in mRNA extracted from A549 and H1299 cells stably knocked down with NSUN2. The amount of RNA loading was detected by methylene blue staining (*bottom*). *E*, methylated-RIP-PCR results for A549 (*left* graph) and H1299 (*right* graph) cells when NSUN2 was knocked down. Each group's relative m5C enrichment of NRF2 mRNA was normalized to the input. *F*, two cysteines of NSUN2 (C271 and C321) were mutated to alanine as shown in the schematic by replacing nucleotides "TG" to "GC". *G*, global mRNA dot blot assays and NRF2 mRNA m5C modification enrichment (*H*) were analyzed after overexpression of WT or mutant NSUN2 in A549 and H1299 cells. Statistical analysis was performed (*right*). *I* and *J*, the schematic of the luciferase reporter vector was cloned with the *NFE2L2* (NM_006164.5) 5′UTR containing either WT or mutant (C-to-A mutation, 55, 56, 58, and 59 sites) m5C sites according to the prediction of m5C Finder database. *J*, the relative luciferase activity of A549 cells transfected with the WT or mutant NFE2L2 5′UTR reporter vectors after NSUN2 knocked down. Data are means ± SD of three independent experiments. *K*, Western blot analysis of NRF2 and NSUN2 expression when NSUN2 WT or mutant plasmid was transfected into A549 cells. *L*, Western blot analysis of NRF2 and NSUN2 expression when NSUN2 WT or mutant plasmid was transfected into NSUN2-knckdown A549 cells, and the cell viability (*M*) and MDA levels (*N*) were conducted after erastin or RSL3 treatment. Data are means ± SD of three independent experiments. ns, not significant; ∗*p* < 0.05; ∗∗*p* < 0.01; ∗∗∗*p* < 0.001. m5C, 5-methylcytosine; NFE2L2, encodes the protein NRF2; NRF2, nuclear factor erythroid 2-related factor 2; NSCLC, non-small cell lung cancer; NSUN2, NOP2/Sun RNA methyltransferase family member 2; RIP, RNA immunoprecipitation.

For us, it was crucial to decipher the mechanism by which NSUN2 regulates NRF2 activation. Firstly, we assessed the NRF2 pre-mRNA levels following *NSUN2* over-expression or knockdown. However, we observed no significant changes (Fig. 7*A*), suggesting that NRF2 might be regulated at the posttranscriptional level. Moreover, after overexpressing NSUN2 in A549 cells and treating them with MG132 and cycloheximide, we did not detect any notable changes in NRF2 protein levels. This indicates that the upregulation of NRF2 protein expression by NSUN2 does not occur by enhancing its protein stability (Fig. 7*B*). Given that NSUN2 functions as an RNA m5C methyltransferase, which can influence RNA stability or translation efficiency, we conducted an RNA immunoprecipitation (RIP) assay with NSCLC cells. The results revealed that NRF2 mRNA is a downstream target of NSUN2 (Fig. 7*C*). In NSCLC cells with *NSUN2* knockdown, the overall mRNA m5C levels displayed a significant decrease, as demonstrated by our dot blot assays (Fig. 7*D*). Similarly, NRF2 mRNA m5C levels were reduced compared to the control, as observed in the methylated RNA immunoprecipitation (MeRIP)-qPCR experiments (Fig. 7*E*). Collectively, our findings validate that NSUN2 operates as an RNA m5C methyltransferase in cancer cells.

NSUN2 is primarily reported to rely on its two cysteine sites, C271 and C321, to exercise its RNA m5C methyltransferase function. The cysteine at the C271 site is essential for RNA binding, while the cysteine at the C321 site forms the core for cytosine methylation catalyzation. To further validate that NSUN2 regulates NRF2 expression through m5C modification, we produced an *NSUN2* overexpression plasmid with mutation sites. We altered the "TG" to "GC" at both NSUN2 C271 and C321 sites, which changed the cysteine to alanine (Fig. 7*F*). Subsequently, we transfected the mutated *NSUN2* plasmid into NSCLC cells and assessed the global m5C levels using a dot blot assay. While a higher m5C level was observed upon overexpressing the NSUN2 WT, there was no significant change in m5C levels when overexpressing the NSUN2 mutant compared to the control group (Fig. 7*G*). The MeRIP-qPCR assay results indicated that NRF2 mRNA has a higher m5C level in NSCLC cells with NSUN2 WT overexpression. In contrast, there were no clear changes, or even diminished levels, in cells with NSUN2 mutant overexpression (Fig. 7*H*). To further substantiate this assertion, we conducted an online prediction by m5C Finder, reconfirming the presence of m5C modification in NRF2 mRNA 5′UTR region and identifying several primary m5C sites. As displayed in Figure 7*I*, we constructed both WT and m5C sites mutated NRF2 mRNA 5′UTR luciferase reporter plasmids. The results revealed that relative luciferase activity diminished in the WT group when *NSUN2* was knocked down. However, the knockdown of NSUN2 significantly decreased the relative luciferase activity of the WT but not of the mutated group (Fig. 7*J*). In sum, our findings suggest that NSUN2 likely modulates NRF2 protein levels by binding to its mRNA in an m5C-dependent manner.

**Supporting Figure S4:**
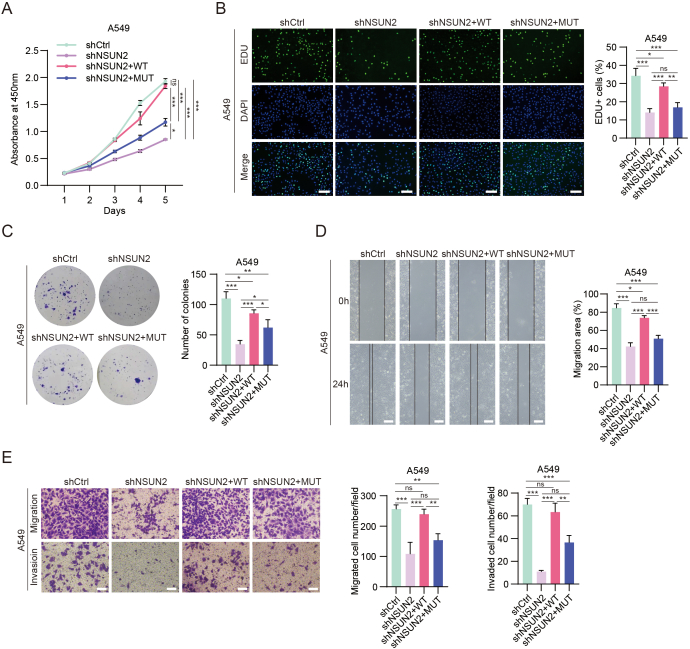
*A*, the proliferation ability of NSUN2-knockdown and rescued by overexpressing WT or methyltransferase lost mutant NSUN2 plasmid A549 cells was determined by CCK8 assay, EdU incorporation assay (*B*), and clonal formation assay (*C*). *D*, the migration and invasion abilities of NSUN2-knockdown and rescued by WT or mutant YBX1 A549 cells were determined by wound healing assay and transwell assays (*E*). Data are mean ± SD of three independent experiments. Scale bar represents 100 μm. ns, not significant; ∗*p* < 0.05; ∗∗*p* < 0.01; ∗∗∗*p* < 0.001.

To figure out whether the m5C methyltransferase of NSUN2 is involved in the modulation of ferroptosis and cancerous phenotypes of NSCLC, we expressed the WT and mutant NSUN2 protein in *NSUN2*-deficient A549 cells. After confirmed the equal expression of the WT and mutant NSUN2 by Western blot (Fig. 7, *K* and *L*), we observed that the expression of NRF2 protein was elevated by overexpressing the WT NSUN2 but not the mutant. Impressively, the mutant NSUN2 almost lost its function in increasing the cell viability (Fig. 7*M*) and decreasing the MDA levels (Fig. 7*N*) compared to the WT. Moreover, it was revealed that the proliferation ability (Fig. S4, *A*–*C*) as well as migration and invasion abilities (Fig. S4, *D* and *E*) of A549 cells transfected with NSUN2 mutant were inferior than those with the NSUN2 WT. Collectively, these findings suggest that NRF2 expression could be modulated by the methyltransferase activity of NSUN2 which lost its oncogenic role in promoting NSCLC progression.

### NSUN2 promotes NRF2 mRNA stability in an m5C-YBX1–dependent manner

**Figure 8:**
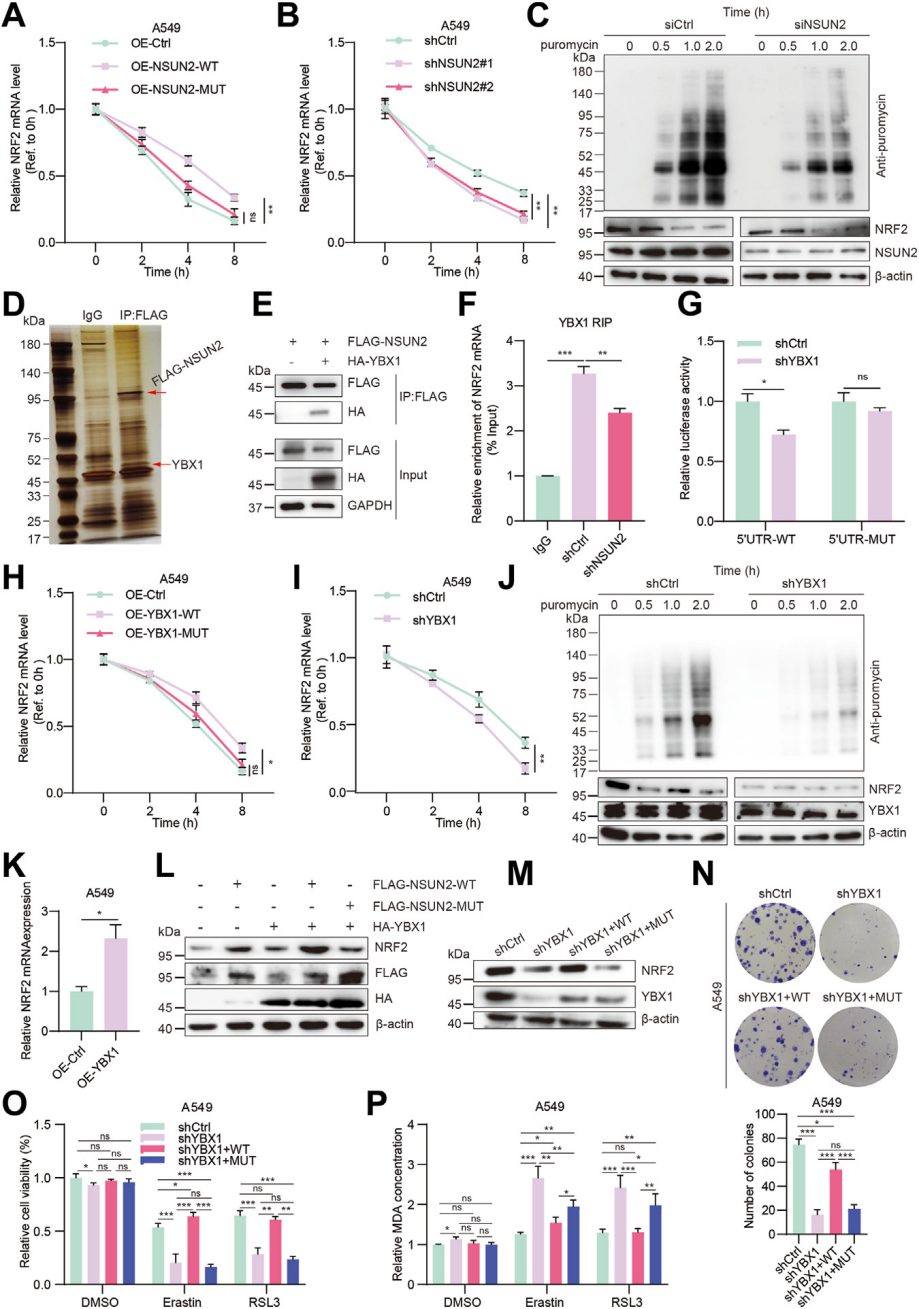
**NSUN2-medicated NRF2 mRNA m5C modification was protected by YBX1.***A* and *B*, the half-life of NRF2 mRNA after overexpressing (*A*) or knocking down (*B*) NSUN2 in A549 cells. *C*, protein expression of A549 cells transfected with siCtrl or siNSUN2 after treatment with puromycin. *D*, YBX1 was found to be associated with NSUN2 as determined by immunoprecipitation and mass spectrometry. *E*, the relationship between YBX1 and NSUN2 was detected by co-immunoprecipitation in A549 cells. *F*, RNA immunoprecipitation of YBX1 and NRF2 mRNA was performed in A549 cells with or without NSUN2 knockdown. IgG was used as the control. *G*, the relative luciferase activity of A549 cells transfected with the WT and mutant form of NFE2L2 5′UTR reporter vectors after YBX1 knockdown. *H* and *I*, the half-life of NRF2 mRNA after overexpressing or knocking down YBX1 in A549 cells. *J*, protein expression of A549 cells transfected with shCtrl or shYBX1 after treatment with puromycin. *K*, NRF2 mRNA levels in A549 cells overexpressing YBX1. *L*, A549 cells were transfected with HA-tagged YBX1 or FLAG-tagged WT and mutant NSUN2 plasmid alone or in combination for 48 h and the protein expression was determined by Western blot. *M*, Western blot analysis of NRF2 and YBX1 protein expression upon overexpression of WT or W65 site mutant YBX1 plasmid in A549 cells pre-treated with shYBX1. *N*, the proliferation ability of A549 cells was determined by clonal formation assay. The statistical analysis graph is below the representative images. *O*, the cell viability and MDA levels (*P*) of YBX1 knockdown A549 cells rescued with WT or mutant YBX1 upregulation. Data are mean ± SD of three independent experiments. shYBX1 means the shYBX1#1. ns, not significant; ∗*p* < 0.05; ∗∗*p* < 0.01; ∗∗∗*p* < 0.001. m5C, 5-methylcytosine; NFE2L2, encodes the protein NRF2; NRF2, nuclear factor erythroid 2-related factor 2; NSUN2, NOP2/Sun RNA methyltransferase family member 2.

The m5C modification of the mRNA 5′UTR region has been reported to influence its stability or translation process. To investigate the impact of NSUN2-mediated m5C modification on the 5′UTR region of NRF2 mRNA, we first conducted an mRNA stability assay. The results revealed that the half-life of NRF2 mRNA increased with the overexpression of NSUN2 WT, but not with the mutant, in comparison to the control (Fig. 8*A*). Conversely, it decreased following *NSUN2* knockdown (Fig. 8*B*). Furthermore, Western blot results from the puromycin incorporation assay indicated that NRF2 expression remained largely unchanged after *NSUN2* knockdown (Fig. 8*C*). These findings suggest that NSUN2 might enhance NRF2 expression by bolstering its mRNA stability, rather than by amplifying its translation process.

**Supporting Figure S5:**
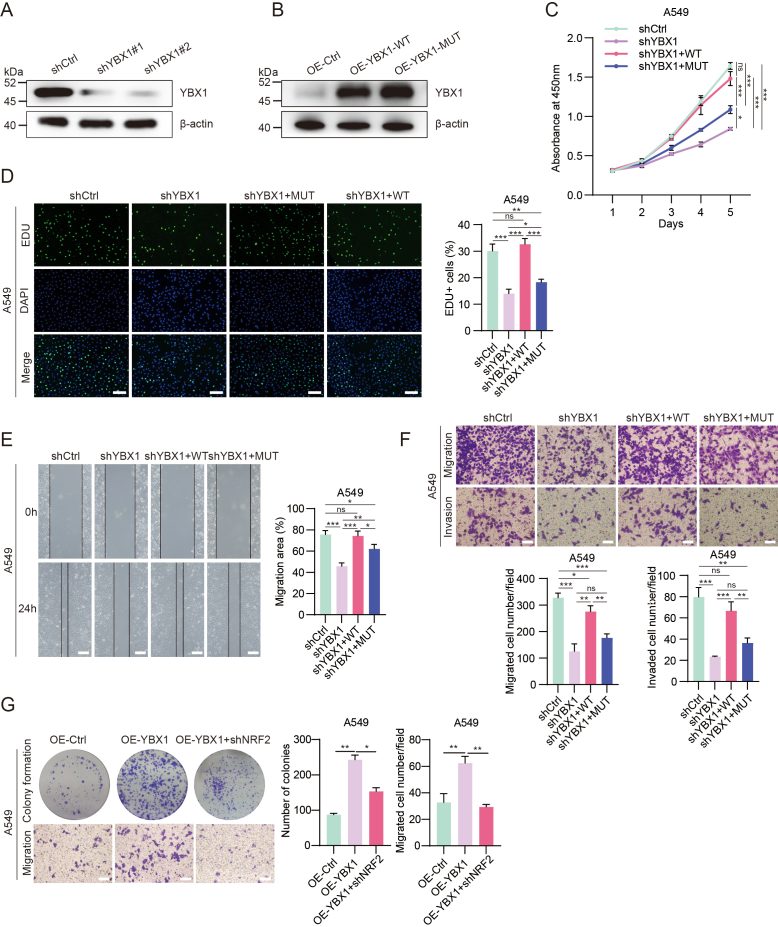
*A*, the western blot of YBX1 knockdown efficiency by shYBX1 in A549 cell. *B*, the western blot of WT (YBX1-WT) and W65 site mutated YBX1 (YBX1-MUT) overexpression efficiency in A549 cell. *C*, the proliferation ability of YBX1 deficient and subsequent rescued by YBX1 WT or mutant overexpression A549 cell was measured by CCK8 assay and EdU incorporation assay (*D*). *E*, the migration and invasion abilities of YBX1 deficient and rescued by WT or mutant YBX1 overexpression A549 cell were measured by wound healing assay and transwell assays (*F*). *G*, the proliferation and migration abilities of YBX1 overexpression with or without and NRF2 deficiency A549 cells were measured by clonal formation assay and transwell assay, respectively. Data are mean ± SD of three independent experiments. Scale bar represents 100 μm ns, not significant; ∗*p* < 0.05; ∗∗*p* < 0.01; ∗∗∗*p* < 0.001.

To elucidate the mechanism by which m5C regulates the elevation of NRF2 protein, we overexpressed flag-tagged NSUN2 in H1299 cells and performed flag affinity purification (Fig. 8*D*). Differentially bound proteins were identified through silver staining, and the immunoprecipitated proteins underwent liquid chromatography-mass spectrometry analysis. Among the proteins identified as interacting with NSUN2, YBX1, a known m5C reader protein, was observed to be relatively abundant. We then further validated the NSUN2–YBX1 interaction using a co-IP assay in H1299 cells (Fig. 8*E*). M5C reader proteins are known to determine the fate of RNA, influencing aspects such as translocation, splicing, stability, translation progression, or interactions with other molecules. YBX1 has been identified as a pivotal m5C reader protein that regulates the stability or translation efficiency of target RNA in various cancer types, and we found that LUAD patients with high *YBX1* expression have a significantly lower overall survival rate in tumor stage III and IV (Fig. S1*B*). To test our hypothesis, we transfected the YBX1 plasmid into NSCLC cells, both with and without *NSUN2* knockdown, and then conducted an RIP experiment. As anticipated, the RIP results showed that the NRF2 mRNA was significantly more abundant in the IP group than in the IgG group, with its levels being markedly lower when *NSUN2* was depleted (Fig. 8*F*). Moreover, after knocking down *YBX1* and confirmed by Western blot (Fig. S5*A*), the relative luciferase activity of NRF2 5′UTR WT decreased and showed no discernible change of the NRF2 5′UTR mutant (Fig. 8*G*). This suggests that YBX1 can bind to NRF2 mRNA and that this binding depends on NSUN2-mediated m5C.

Our next step was to explore the impact of YBX1 on NRF2 m5C modification. As depicted in Figure 8, *H* and *I*, the half-life of NRF2 mRNA increased with overexpression of either NSUN2 or YBX1 WT, but not their mutated forms, it decreased upon the knockdown of NSUN2 or YBX1. The WT and W65A site mutant YBX1 have a similar expression level as demonstrated by our Western blot (Fig. S5*B*). The puromycin incorporation assay, however, indicated that NRF2 translation progression was not significantly affected by *YBX1* knockdown (Fig. 8*J*). Additionally, upon *YBX1* overexpression, we observed an upregulation in the level of NRF2 mRNA (Fig. 8*K*), and only the NSUN2 WT, not its mutant form, could synergistically enhance NRF2 protein expression upon *YBX1* overexpression (Fig. 8*L*). Our Western blot results further substantiated that the enhancement of NRF2 expression is contingent upon YBX1's reader function; the W65-mutated YBX1 lost its ability to promote NRF2 protein expression when *YBX1* was knocked down (Fig. 8*M*). Our functional experiments indicate that the mutant YBX1 lost its ability in promoting cell proliferation as demonstrated by CCK8 assay (Fig. S5*C*), EdU incorporation assay (Fig. S5*D*), and clonal formation assay (Fig. 8*N*), as well as the migration and invasion abilities as demonstrated by wound healing assay (Fig. S5*E*) and transwell assays (Fig. S5*F*), following with lower cell viability (Fig. 8*O*) and higher MDA concentrations (Fig. 8*P*), when compared to the WT YBX1 in *YBX1*-knockdown A549 cells. What’s more, our clonal formation and transwell assay results (Fig. S5*G*) demonstrated that WT YBX1 overexpression augmented the proliferation and migration capabilities of A549 cells. This effect was neutralized by subsequent NRF2 knockdown. These findings suggest that YBX1 plays a role in NSUN2-mediated NRF2 mRNA m5C formation, contributing to the increase in NRF2 mRNA stability without enhancing its translation efficiency. This implies that the NSUN2–m5C–YBX1 axis promotes NRF2 expression in NSCLC cells.

**Figure 9:**
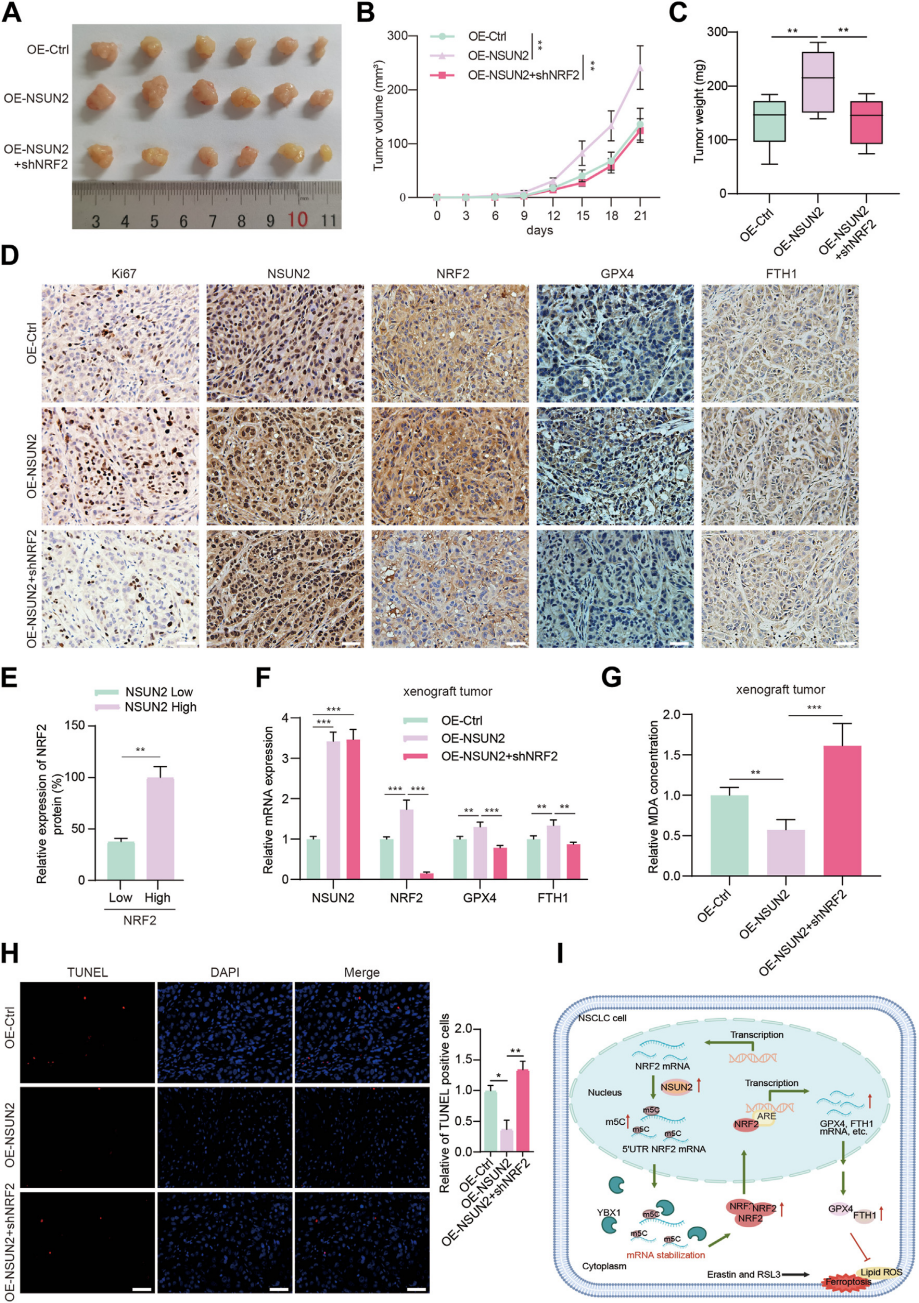
**NSUN2 facilitates tumorigenesis of NSCLC cells in a NRF2-dependent manner *in vivo*.***A*, images of tumors formed in nude mice bearing control A549 cells and NSUN2-overexpression cells with or without NRF2 inhibition. *B*, tumor volumes were monitored every 3 days and tumor growth curves were shown. *C*, the weight of xenograft tumors between different groups were measured at the endpoint of experiment. *D*, representative images of IHC images of Ki-67, NSUN2 and NRF2, GPX4 and FTH1 staining in serial sections of xenograft tumors isolated from subcutaneous models. Scale bar represents 50 μm. *E*, the correlation of NRF2 protein and NSUN2 protein expression in IHC analysis from xenograft tumors. *F*, the mRNA levels of NSUN2, NRF2, GPX4, and FTH1 were determined by RT-qPCR in xenograft tumors of each group. *G*, the MDA levels in xenograft tumors of each group were measured and analyzed. *H*, TUNEL-positive (*red*) cells were detected in xenograft tumor tissues formed in nude mice by TUNEL staining and the statistical results. The data are expressed as mean ± SD (n = 6). Scale bar represents 50 μm. *I*, working model of NSUN2–YBX1–NRF2 axis in ferroptosis resistance. The m5C modification of NRF2 mRNA medicated by NSUN2 increased its stability, which is recognized and protected by YBX1, thereby upregulating NRF2 expression and promoting NSCLC resistance to ferroptosis. ∗*p* < 0.05, ∗∗*p* < 0.01, ∗∗∗*p* < 0.001. IHC, immunohistochemistry; m5C, 5-methylcytosine; NRF2, nuclear factor erythroid 2-related factor 2; NSCLC, non-small cell lung cancer; NSUN2, NOP2/Sun RNA methyltransferase family member 2; RT-qPCR, real time quantitative PCR.

To investigate the biological function of NSUN2 *in vivo*, we chose to use immunodeficient mouse models for the subject of our research. We subcutaneously injected control cells or *NSUN2*-overexpressing A549 cells, with or without NRF2 inhibition, into the armpits of nude mice in different groups. After a 3-week observation period, tumors were harvested for further analysis. Compared to the control group, the mice that received OE-NSUN2 cell implants displayed accelerated tumor formation, along with a significant increase in tumor growth and weight. On the contrary, cells deficient in NRF2 hindered these effects (Fig. 9, *A*–*C*). Additionally, the HE staining of *NSUN2* overexpression xenograft tumors exhibit increased tumor malignancy state, and the immunohistochemistry staining also verified that the tumors promoting growth that emerged from A549 cells with *NSUN2* overexpression exhibited heightened levels of Ki-67 and NRF2 as well as GPX4 and FTH1 (Fig. 9*D*). NRF2 protein expression forms a positive correlation with NSUN2 (Fig. 9*E*). Further, it was obviously that the subcutaneous tumors in NSUN2 high group have an average lower MDA concentration but higher mRNA levels of *NRF2*, *GPX4*, and *FTH1*, which means ferroptosis inhibition when compared to control or NSUN2-high but NRF2 deficiency group. What’s more, the TUNEL staining results showed that NSUN2 could significantly decrease the apoptotic levels of xenograft tumor tissues; silencing *NFE2L2* substantiality reversed this effect (Fig. 9*H*). Together, these *in vivo* findings, supporting the above *in vitro* results, establish the cancer-promoting function of NSUN2 in the advancement of NSCLC.

## Discussion

Lung cancer remains the leading cause of cancer-related deaths worldwide. There are two primary types of lung cancer: NSCLC and small-cell lung cancer, accounting for 80% and 20% of all lung cancers, respectively (2). NSCLC is the most malignant subtype of lung cancer. Its incidence and mortality have significantly increased over the past few decades. Approximately, 70% of NSCLC patients are diagnosed with metastases, leading to a 5-year survival rate of only 15% (32, 33). Given the rising incidence of NSCLC, it is essential to identify molecules that regulate lung cancer growth, metastasis, and survival. While studying the underlying molecular mechanisms can pave the way for new treatment approaches, the molecular pathogenesis of this disease is still not well understood. Hence, there is a pressing need to identify new markers and therapeutic targets for treating NSCLC. In this context, our data proposes a model where NSUN2 regulates the ferroptosis process in lung cancer cells through an m5C-YBX1–dependent manner and acts as a promoter in advancing lung cancer progression (Fig. 9*I*).

Ferroptosis plays a pivotal role in the regulation of NSCLC, and modulating ferroptosis might emerge as a potential therapeutic strategy for NSCLC treatment (34). While NSUN2 has been reported to influence cancer development, chemoresistance, and oxidative stress (6), there has been limited research on its role in ferroptosis. In this study, we observed that NSUN2 was highly expressed in NSCLC cells, and its elevated expression was associated with a poor prognosis in NSCLC. Furthermore, we discovered that inhibiting *NSUN2* heightened the sensitivity of NSCLC cells to both erastin and RSL3-induced ferroptosis, a shift mediated by the downregulation of NRF2 protein expression. NRF2 is renowned for its integral role in managing oxidative stress and ferroptosis. When faced with oxidative stress, KEAP1 disengages from NRF2, facilitating NRF2's migration into the nucleus, where it triggers the expression of compensatory genes, including *GPX4* and *FTH*, both of which are involved in ferroptosis (35, 36). Given the pivotal position NRF2 occupies in chemoprevention and cancer therapy, the activation of the NRF2/KEAP1 signaling pathway in cancer cells often results in chemoresistance, neutralizing drug-induced oxidative stress, and shielding cancer cells from drug-mediated cell death (37). Interestingly, in our studies, despite the overexpression of *NSUN2*, there were no discernible changes in NRF2 protein stability when cells were treated with the translation inhibitor cycloheximide or the proteasome pathway inhibitor MG132 over the specified duration. Consequently, our findings shed light on a novel, noncanonical pathway where NRF2 activation, modulated by NSUN2, operates independently of the KEAP1-mediated mechanism.

RNA methylation, which potentially plays a vital role in regulating gene expression, splicing, RNA editing, RNA stability, and controlling mRNA lifespan and degradation, is a pivotal facet of epigenetics research (38). NSUN2-mediated mRNA m5C modification has been reported to primarily influence mRNA stability or translation efficiency across various cancers. Our current study observed that NSUN2 protein expression is positively correlated with NRF2 *in vitro* and *in vivo*. Consequently, we were keen on examining the m5C modification of NRF2 mRNA. To decipher the relationship between NRF2 and m5C modification, our RIP-qPCR and MeRIP-qPCR assays substantiated that NSUN2 could specifically interact with and methylate NRF2 mRNA. Moreover, guided by the online m5C site prediction, our luciferase reporter assay reinforced this prediction; the relative fluorescence intensity of the NRF2 mRNA 5′UTR WT reporter group diminished post-*NSUN2* knockdown, in contrast to the m5C site mutant reporter group. Following this, we delved into the impact of the 5′UTR m5C modification on NRF2 mRNA. Employing actinomycin D or puromycin treatments, we ascertained that the m5C modification of NRF2 mRNA by NSUN2 bolstered its stability without influencing the translation process. Additionally, subsequent experiments corroborated the involvement of the m5C reader YBX1 in NRF2 regulation. These findings suggest that NSUN2 amplifies NRF2 mRNA stability through m5C deposition, elevating its expression in NSCLC.

The ferroptosis-cell death mechanism is pivotal for anticancer therapy, and efficient anticancer drugs can induce ferroptosis. It represents one of the primary defense mechanisms against ferroptosis, regulated by the NRF2 signaling pathway. Many inhibitors targeting NRF2, such as Brusatol and Luteolin, have been employed for cancer treatment (39, 40). Several mechanisms for NRF2 activation in cancer have been reported: (1) mutations in KEAP1, CUL3, or NRF2; (2) epigenetic silencing of *KEAP1*; (3) disruption of interactions between KEAP1 and NRF2 proteins, such as P21 and P62 (41). Our study uncovered a novel KEAP1-independent mechanism for NRF2 activation, suggesting the theoretical feasibility of developing a drug targeting NSUN2 to reduce the overall m5C level and malignant phenotype of cancer cells. Moreover, an adjuvant drug could potentially block NRF2's mRNA hypermethylation, leading to decreased NRF2 expression levels and thus heightening the sensitivity of cancer cells to conventional chemotherapeutic agents. However, a more comprehensive understanding of how NSUN2 regulates NRF2-mediated ferroptosis is needed for future research. In conclusion, our findings point to a potential therapeutic strategy for combatting this devastating disease, suggesting that NSUN2 could serve as a prognostic biomarker and therapeutic target in future lung cancer treatments.

## Experimental procedures

### Patients and clinical samples

Surgical specimens from 120 lung adenocarcinoma patients were obtained postoperatively from The First Affiliated Hospital of Zhengzhou University. All patients were diagnosed with lung cancer based on pathological and/or cytological evidence. The tissues were collected before any chemotherapy or radiotherapy treatments. Some tissues were immediately frozen and stored at −80 °C for subsequent Western blot analysis. The remaining tissues were fixed with 4% paraformaldehyde for IHC analysis. Each patient provided informed consent, and the study was approved by the ethics committee of The First Affiliated Hospital of Zhengzhou University.

### Immunohistochemistry

Paraffin-coated tissues were heated at 60 °C for 4 h and then deparaffinized with BioDewax and Clear Solution (ServiceBio). After deparaffinization, heat-induced antigen retrieval was performed. The tissues were subsequently blocked with 3% BSA at room temperature for 30 min. They were then incubated with the primary antibody overnight at 4 °C. The next day, the slides were treated with horseradish peroxidase–conjugated secondary antibodies for 1 h at room temperature and subsequently exposed to DAB. Slides were then stained with hematoxylin, followed by decolorization using ethanol and hydrochloric acid, and finally fixed and dried. Images were captured under a microscope.

### TUNEL staining

The TUNEL staining kit (Beyotime) was used to detect the apoptosis rate in each group of xenograft tumors, following the manufacturer's instructions. Nuclei were stained with DAPI, and TUNEL-labeled cells (red) were manually counted in three randomly chosen fields of view from each well to calculate the percentages of stained cells.

### Cell culture

Human NSCLC cell lines, A549 and H1299, were cultured in DMEM and RPMI-1640, respectively, supplemented with 10% fetal bovine serum and 1% penicillin/streptomycin, in a 5% CO2 environment at 37 °C. For sensitivity analysis of ferroptosis inducers, A549 and H1299 cells were treated with 10 μM erastin (Cat. No. S7242, Selleck) or 4 μM RSL3 (Cat. No. S8155, Selleck). All experiments were performed with mycoplasma-free cells. The cells were purchased from Procell Life Science &Technology and validated by short tandem repeat profiling.

### Cell transfection, antibodies, and RT-qPCR primers

The pLV-FLAG-neg (OE-Ctrl) and pLV-FLAG-NSUN2 (OE-NSUN2) plasmids were used for NSUN2 overexpression, while the pLV-sh-neg (shCtrl), pLV-shNSNUN2#1, and pLV-shNSUN2#2 plasmids were used for NSUN2 knockdown. The shYBX1 plasmid was employed for YBX1 knockdown. The sequence for shNSUN2#1 is 5′-CAGTGGAAGGTAATGACGAAA-3′, for shNSUN2#2, it is 5′-TGACGTGTCCCATCGTCTTAT-3′ and for shYBX1#1, it is 5′-CCACGCAATTACCAGCAAA-3′, for shYBX1#2, it is AGCAGACCGTAACCATTATAG. The siRNA for NSUN2 was sourced from RiboBio, with transfection being carried out using Lipofectamine 3000 (Invitrogen). NSUN2 KO plasmid was derived from WZ Bioscience Inc, gRNA: TGTTCTCCTTGACGATCTCG, and was packed by lentivirus for cell infections.

The antibodies used in this study include anti-NSUN2 (Cat. No. 20854-1-AP, WB: 1:1000, IHC: 1:200, Proteintech), anti-FLAG (Cat. No. AF2852, WB 1:1000, Beyotime), anti-NRF2 (Cat. No. CY5136, WB 1:1000, IHC 1:100, Abways), anti-HA (Cat. No. 81290-1-RR, WB 1:5000, Proteintech), anti-β-actin (Cat. No. 81115-1-RR, WB 1:2000, Proteintech), anti-YBX1 (Cat. No. CY5462, WB 1:1000, Abways), anti-Rabbit IgG (Cat. No. 30000-0-AP, Proteintech), anti-mouse IgG (Cat. No. B900620, Proteintech), anti-GPX4 (Cat. No. CY6959, WB 1:800, IHC 1:100, Abways), anti-FTH1 (Cat. No. CY7085, WB 1:800, IHC 1:100, Abways).

The primers used in this article are as follows:

NSUN2 F: 5′-AGTTCATGGACGCTCTCAGG-3′

R:5′-ACTTTCTGACCGTCCACCTC-3′

NFE2L2 F: 5′-GCCAACTACTCCCAGGTTGC-3′

R: 5′-GTGACTGAAACGTAGCCGAAG-3′

pre-NFE2L2#1 F: 5′-CAGAGCGGCTTTGTCTTTGG-3′

R: 5′-ACTCGCAACTCTTACCCTTGA-3′

pre-NFE2L2#2 F: 5′-AGGAAGGATTGGAGGGTGCT-3′

R: 5′-TCAGAGTTCCCAGATCAGACG-3′

GPX4 F: 5′-GAGCCAGGGAGTAACGAAGAG-3′

R: 5′-TGGTGAAGTTCCACTTGATGG-3′

FTH1 F: 5′-GACCCCCATTTGTGTGACTTC-3′

R: 5′-ATTATCACTGTCTCCCAGGGT-3′

β-actin F: 5′-GAGAAAATCTGGCACCACACC-3′

R: 5′-GGATAGCACAGCCTGGATAGCAA-3′

YBX1 F: 5′-GACAAAAGCAGCCGATCCAC-3′

R: 5′-TGTTGGATGACTAAACCGGATG-3′

### EdU, CCK 8, and colony formation assays

The BeyoClick EdU Cell Proliferation Kit with Alexa Fluor 488 (Beyotime) was utilized for EdU assays and carried out as the provided instructions. Cell images were captured using an Inverted Fluorescence Microscope (Olympus). For CCK-8 assays, 1500 cells were seeded per well in 96-well plates. Subsequently, 100 μl of complete medium and 10 μl of the CCK-8 reagent (Dojindo) were added to each well and incubated at 37 °C for 2 h, after which absorbance was measured at 450 nm. Cells were seeded into 6-well plates at 800 cells per well for colony formation assays and then incubated at 37 °C for 2 weeks. Afterward, cells were fixed and stained with 4% crystal violet, with colonies subsequently being counted. These counts were based on the averages from three independent experiments.

### Cell cycle assay

The Cell Cycle Assay Kit - PI/RNase Staining was used to detect the cell cycle after the modification of NSUN2 expression according to the instructions. Briefly, after collected by trypsin and washed by cold PBS for three times in tube, the cell was treated with 70% ethanol for 2 h at 4 °C. Then, the cell was centrifugated and washed with cold PBS and stained with 0.5 ml work solution for 1 h at 4 °C protected from light. After that, the cell cycle was measured by flow cytometry.

### Wound healing and transwell assays

For wound-healing assays, 5 × 105 cells were seeded into 6-well plates and allowed to adhere for 24 h. Afterward, gaps were created, and the cells were further incubated in a serum-free medium for an additional 24 h. The percentage of migrated cell area was then calculated and normalized to the 0-h time point. For transwell assays, lung cancer cells from various transfection groups were suspended in a serum-free medium and placed into the upper chamber of the transwell insert with 100 μl of cell suspension. For invasion assays, the insert was pre-coated with Matrigel. The lower chamber of the transwell setup was filled with medium enriched with 20% FBS, serving as a chemoattractant. After 24 h of incubation, the transwell inserts were rinsed with PBS, fixed with 4% formaldehyde for 10 min, and stained with 0.1% crystal violet for 30 min.

### Cell viability, MDA, and GSH assays

Lung cancer cells were seeded into 96-well plates (5000 cells per well) and treated with chemical compounds, erastin or RSL3, for cell viability assays. After a 12-h treatment, cell viability was assessed using the CCK8 cytotoxicity assay, measuring absorbance at 450 nm. For MDA assays, the supernatant from the whole cell lysate of transfected cells in 10 cm plates was collected using RIPA lysis buffer. The protein concentration in the supernatant was determined using the BCA assay kit. Subsequently, 100 μl of supernatant was mixed with the MDA working solution and heated at 65 °C for 15 min, following the manufacturer's instructions (Cat. No. S0131S, Beyotime). The mixture was then centrifuged at 1000*g* for 10 min, and 200 μl of the resulting supernatant was transferred to 96-well plates for absorbance measurement at 532 nm. GSH concentration in cell lysates was measured using a Glutathione Assay Kit (S0056, Beyotime) according to the manufacturer’s instructions. Results were calculated using the standard curve and normalized to the control group.

### Assessment of lipid peroxidation with BODIPY and flow cytometry analysis

Cells from the NSUN2 knockdown or control groups were seeded in 6-well plates and cultured overnight. They were then treated with either DMSO or erastin for 12 h. Subsequently, the cells were harvested, washed with precooled Hanks' Balanced Salt Solution, and then incubated with the working solution, following the protocol provided by the Lipid Peroxidation Probe -BDP 581/591 C11 kit (DOJINDO).

### RNA-seq and analysis

RNA was extracted from A549 cells stably overexpressing NSUN2 using RNAiso Plus (Takara) and then assessed for both RNA integrity and total quantity with the 2100 bioanalyzer (Agilent), as previously described (42). The RNA sequencing library was subsequently prepared and sequenced on the Illumina HiSeq 6000 platform (Illumina) by Novogene (Beijing). DEGs were identified using the DESeq2 R package (version 3.0.3) with a significance threshold of *p*-value ≤ 0.05 and |log2FoldChange| ≥ 0.3. The cluster Profiler R package (version 3.0.3) was used to perform gene ontology and Kyoto Encyclopedia of Genes and Genomes enrichment analysis on the DEGs and to generate the heatmap.

### RIP-qPCR and MeRIP-qPCR

The RIP-qPCR procedure was carried out according to the PureBindingRNA Immunoprecipitation Kit protocol (Cat. No. P0101, GENESEED). For meRIP-qPCR, roughly 100 μg of total RNA extracted from NSCLC cells was incubated with magnetic beads coated with 5 μg anti-m5C antibody (Abcam, ab214727) or anti-IgG antibody (Abcam, ab172730) for 6 h at 4 °C, forming magnetic bead–antibody–RNA complexes. RNA was subsequently eluted from these complexes using proteinase K digestion buffer and extracted using a buffer composed of phenol: chloroform: isoamyl alcohol in a ratio of 125:24:1. The RNA levels were further analyzed using RT-qPCR.

### RNA dot blot

After extracting total RNA from NSCLC cells, mRNA was isolated using the mRNA Isolation System IV kit (Poly Tract). Concentrations of 100, 200, and 400 ng/μl of mRNA were denatured with DEPC water at 95 °C for 5 min and then transferred onto an Amersham Hybond N+ membrane (GE Healthcare). The membrane was cross-linked using UV light for 5 min and subsequently stained with 0.02% methylene blue (Solarbio). Following this, the membrane was blocked with 5% nonfat dry milk in 1xTBSTw for 1 h at room temperature and incubated with anti-m5C antibody (1:1000, Abcam, ab214727) overnight at 4 °C. After incubation with the secondary antibody, the membrane was visualized using an imaging system.

### RNA stability

Stably transfected NSCLC cells were treated with actinomycin D (5 μg/ml, Med ChemExpress) for 0, 2, 4, and 8 h. Subsequently, total RNA was extracted using Trizol reagent (Takara). The abundance of mRNA levels at each indicated time point was assessed by RT-qPCR and normalized to the 0-h time point.

### Luciferase reporter assay

The luciferase assay was conducted according to the manufacturer's instructions, using reporter lysis buffer and luciferase assay reagent. Both WT and NSUN2-MUT cells were transfected with pmiRGLO, pmiRGLO-WT-5′UTR, and pmiRGLO-MUT-5′UTR in a 6-well plate. After 12 h of transfection, the cells were transferred to 96-well plates and incubated for an additional 24 h. The Dual-Glo Luciferase Assay system (Promega) was used to analyze the results. Renilla Luciferase (R-luc) served as a normalization control for firefly luciferase (F-luc) activity, facilitating the assessment of reporter translation efficiency.

### Protein translation assay

Protein translation levels *in vitro* were assessed using the puromycin incorporation assay and an anti-puromycin antibody (Cat. No. EQ0001, 1:1000, Kerafast). Cells were treated with 200 ng/ml puromycin (BL528A, Biosharp) for the specified durations. Subsequently, cells were lysed, and protein expression was analyzed by Western blot, with β-actin serving as a reference.

### Tumor xenograft model

Female Balb/c nude mice, aged 4 to 5 weeks, were purchased from GemPharmatech. The entire animal experiment received approval from the Animal Experimentation Ethics Committee of Zhengzhou University. Four mice per cage were housed in a specific pathogen-free environment with consistent temperature and humidity. After a 3-days observation and acclimatization period, each mouse was subcutaneously injected with 100 μl containing 10^6^ stably transfected A549 cells. Tumor volume was consistently measured and recorded. Roughly 3 weeks later, the tumors were harvested, and their weights were noted.

### Statistical analysis

Statistical analysis was conducted using GraphPad Prism 9.0. Data are presented as the mean ± SD. A two-tailed Student's *t* test was utilized to compare means between groups as specified. Overall survival was assessed using the Kaplan-Meier method, and *p*-values were determined with the log-rank test. A value of ∗*p* < 0.05 was considered significant.

## Data availability

All data supporting the results of this study are available in the article when requested.

## Supporting information

This article contains supporting information (43, 44).

## Conflict of interest

The authors declare that they have no conflicts of interests with the contents of this article.
